# Global Analysis of *WOX* Transcription Factor Gene Family in *Brassica napus* Reveals Their Stress- and Hormone-Responsive Patterns

**DOI:** 10.3390/ijms19113470

**Published:** 2018-11-05

**Authors:** Mang-Mang Wang, Ming-Ming Liu, Feng Ran, Peng-Cheng Guo, Yun-Zhuo Ke, Yun-Wen Wu, Jing Wen, Peng-Feng Li, Jia-Na Li, Hai Du

**Affiliations:** 1 College of Agronomy and Biotechnology, Southwest University, Chongqing 400715, China; mangmangwang16@126.com (M.-M.W.); mingmingliu95@163.com (M.-M.L.); fengran91@126.com (F.R.); gpc930813@163.com (P.-C.G.); kyz2014@email.swu.edu.cn (Y.-Z.K.); wuyunwen97@163.com (Y.-W.W.); luckywenjing93@126.com (J.W.); pengfengli17@126.com (P.-F.L.); ljn1950@swu.edu.cn (J.-N.L.); 2 Academy of Agricultural Sciences, Southwest University, Chongqing 400715, China

**Keywords:** WOX family, *Brassica napus* L., phylogenetic analysis, expression analysis

## Abstract

The plant-specific *WUSCHEL-related homeobox* (*WOX*) transcription factor gene family is important for plant growth and development but little studied in oil crops. We identified and characterized 58 putative *WOX* genes in *Brassica napus* (*BnWOX*s), which were divided into three major clades and nine subclades based on the gene structure and conserved motifs. Collinearity analysis revealed that most *BnWOX*s were the products of allopolyploidization and segmental duplication events. Gene structure analysis indicated that introns/exons and protein motifs were conserved in each subclade and RNA sequencing revealed that *BnWOX*s had narrow expression profiles in major tissues and/or organs across different developmental stages. The expression pattern of each clade was highly conserved and similar to that of the sister and orthologous pairs from *Brassica rapa* and *Brassica oleracea*. Quantitative real-time polymerase chain reaction showed that members of the WOX4 subclade were induced in seedling roots by abiotic and hormone stresses, indicating their contribution to root development and abiotic stress responses. 463 proteins were predicted to interact with Bn*WOX*s, including peptides regulating stem cell homeostasis in meristems. This study provides insights into the evolution and expression of the WOX gene family in *B. napus* and will be useful in future gene function research.

## 1. Introduction

The WUSCHEL-related homeobox (WOX) family is a large group of transcription factors (TFs), specifically found in plants and characterized by the presence of 60–66 amino acids that constitute a DNA-binding domain, called a homeodomain (HB domain) [1]. The HB domain is highly conserved and important for the function of WOX protein family members, which are also characterized by the presence of a helix–loop–helix–turn–helix secondary structure.

Previous reports have shown that *WOX* genes play a wide variety of roles in plant development and growth processes such as embryonic patterning, stem cell maintenance and organ formation [2,3]. In *Arabidopsis thaliana*, there are at least 15 *WOX* genes (*AtWOXs*), classified into three clades: an ancient clade (*WOX10*, *WOX13* and *WOX14*), intermediate clade (*WOX8*, *WOX9*, *WOX11* and *WOX12*) and modern clade (*WUS* and *WOX1–7*) [2,4,5]. The functions of *AtWOX*s have been well studied and shown to be generally distinct in members of different clades and conserved in members of the same clade. For example, members of the modern clade maintain apical stem cells [6,7] and members of the intermediate clade mainly regulate zygote development and early embryo morphogenesis [8,9], while members of the ancient clade regulate root development [10]. The functions of *WOX*s in many other plants are also conserved and similar to those of their *Arabidopsis* homologs. For instance, in rice (*Oryza sativa*) and maize (*Zea mays*), the orthologs of *AtWOX5* (*OsWOX5* and *ZmWOX5*) play similar roles in the root meristem development [11,12]; in Norway spruce (*Picea abies*), *PaWOX8* and *PaWOX9* are orthologs of the members of the AtWOX8/9 subcladeand determine the cell fate during early embryo patterning [13]; and *PtWUS* in *Populus trichocarpa* corresponds to *AtWUS,* which is involved in shoot apical meristem (SAM) development.

*Brassica napus* L. (*B. napus*) has a high economic value because it is one of the main commercial sources of cooking oil. *B. napus* is an allotetraploid species (AnAnCnCn; *n* = 19), which originated from the recent hybridization of two base diploid genomes of *Brassica rapa* L. (*B. rapa*; AnAn; *n* = 10) and *Brassica oleracea* L. (*B. oleracea*; CnCn; *n* = 9) [14]. Currently, there is a lack of detailed genomic information on the WOX gene family of *B. napus*, but it is of interest to explore the evolution and expression mechanisms of *WOX* genes (*WOXs*) between the *Brassica* An and Cn (BnAn and BnCn) sub-genomes [15]. Furthermore, understanding the structural relationships between *Arabidopsis* and *B. napu*s would facilitate the prediction of *B. napu*s *WOX* genes that remain uncharacterized.

In the present study, we performed a comprehensive genome-wide analysis of the WOX gene family in *B. napus* and identified 58 putative genes (*BnWOXs*) with open reading frames (ORFs). Phylogenetic analysis classified these *BnWOX*s into three clades, characterized by the integration of sequence features, chromosomal location, phylogenetic relationship, subcellular localization, interacting proteins, collinearity and expression patterns. In addition, quantitative real-time polymerase chain reaction (qRT-PCR) analysis showed that members of the WOX4 subclade were induced by abiotic stresses or hormones in seedling roots, suggesting their function in stress tolerance. Overall, our study will serve as a foundation for future research on the functional roles of *WOX*s in *B. napus*.

## 2. Results

### 2.1. Identification of 58 BnWOX Genes and Their Physicochemical Properties

To identify the WOX-encoding genes in the *B. napus* genome, a preliminary BLASTP search was performed using the HB domain sequences of known *Arabidopsis* WOX proteins as queries. In each case, a large number of deduced amino acid sequences (>50 candidates) containing WOX or WOX-like repeats were obtained. Only hits with E-values of <1.0 were considered as members of the WOX gene family. The redundant candidate sequences were discarded from our data set, according to their chromosomal locations. We were then able to identify 58 typical, non-redundant *WOX* genes in the *B. napus* genome; those had complete ORF regions and encoded proteins with typical WOX features, which we verified using PROSITE (http://www.expasy.org/tools/scanprosite/). To distinguish these genes, we provisionally named them *BnWOX1* to *BnWOX58* based on their order on the corresponding chromosomes (Table 1). We also identified 27 and 30 non-redundant *WOX* genes in *B. rapa* (*BrWOXs*) and *B. oleracea* (*BoWOXs*), respectively, using the same method (Table S1). Physicochemical property analysis showed that the corresponding BnWOX proteins (BnWOXs) varied in length from 121 to 390 amino acids; their molecular weight ranged from 13.96 to 71.82 kDa and the isoelectric points were 5.08–9.58. Subcellular localization analysis demonstrated that all 58 proteins were located in the nucleus (Table 1).

### 2.2. Phylogenetic Analysis of the BnWOX Gene Family

To determine the evolutionary relationships of the *BnWOX* gene family with those of *Arabidopsis* and the *B. napus* ancestor species, we constructed neighbor-joining (NJ) and maximum-likelihood (ML) phylogenetic trees of 130 WOX proteins, from *B. napus* (58), *B. rapa* (27), *B. oleracea* (30) and *Arabidopsis* (15), based on the multi-alignment of their HB domains using MEGA 7.0 [16] and PhyML 3.0 [17], respectively.

**Figure 1:**
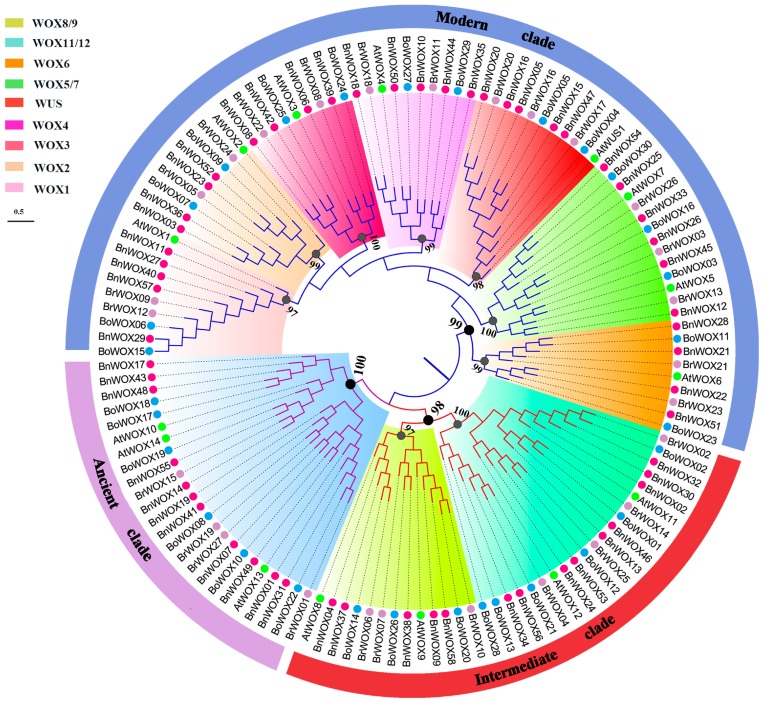
Phylogenetic relationships of 130 *WOX* genes. The phylogenetic tree was generated from the alignment of 130 WOX protein homeodomain sequences, with 1000 bootstrap replicates. The *WOX* genes from *Arabidopsis* (At: 15), *B. rapa* (Br: 27), *B. oleracea* (Bo: 30) and *B. napus* (Bn: 58) are shown as green, purple, blue and red dots, respectively. The outer circle represents three clades, marked in purple, blue and brown colors. Nine subclades were identified and are marked in different background colors; bootstrap values are shown near the nodes.

Our results showed that the NJ and ML tree topologies were highly congruent (Figure 1 and Figure S1). In the phylogenetic trees, the 130 WOX members clustered into three main clades: modern, intermediate and ancient clades (Figure 1). The number of *WOX* genes in the modern clade (72 genes) was greater than that in the ancient (24 genes) and intermediate (34 genes) clades, indicating the gene expansion in higher plants. Our data were consistent with those of previous reports, which indicated that the WOX gene family was chronologically divided into three clades (i.e., ancient, intermediate and modern/WUS clades) [4]. In each clade, the numbers of genes from these four species were usually different and each *Arabidopsis WOX* gene (*AtWOX*) generally had more than one ortholog. Thus, the modern clade consisted of 33 *BnWOX*s, 15 *BoWOX*s, 16 *BrWOX*s and 8 *AtWOX*s; the intermediate clade included 14 *BnWOX*s, 9 *BoWOX*s, 7 *BrWOX*s and 4 *AtWOX*s; and the ancient clade had 11 *BnWOX*s, 6 *BoWOX*s, 7 *BrWOX*s and 3 *AtWOX*s. This distribution showed that the number of genes constantly increased with the evolution of the gene family.

We further divided the candidate *WOX* genes into nine subclades: WUS, WOX1, WOX2, WOX3, WOX4, WOX5/7, WOX6, WOX11/12 and WOX8/9, based on the bootstrap values and the topology of the phylogenetic tree. There were seven subclades in the modern clade and two in the intermediate clade (Figure 1). In the ancient clade, only homologs of *WOX13* were found in non-Brassicaceae species [3,18,19,20]; however, we found that homologs of *WOX10/14* existed in all of the four Brassicaceae, which indicates that *WOX10/14* may be unique to Brassicaceae [21]. In addition, the *WOX1/WOX6* homologs were divided into two subclades (WOX1 and WOX6), with high bootstrap values.

### 2.3. Sequence Analysis of B. napus WOX Domains

**Figure 2:**
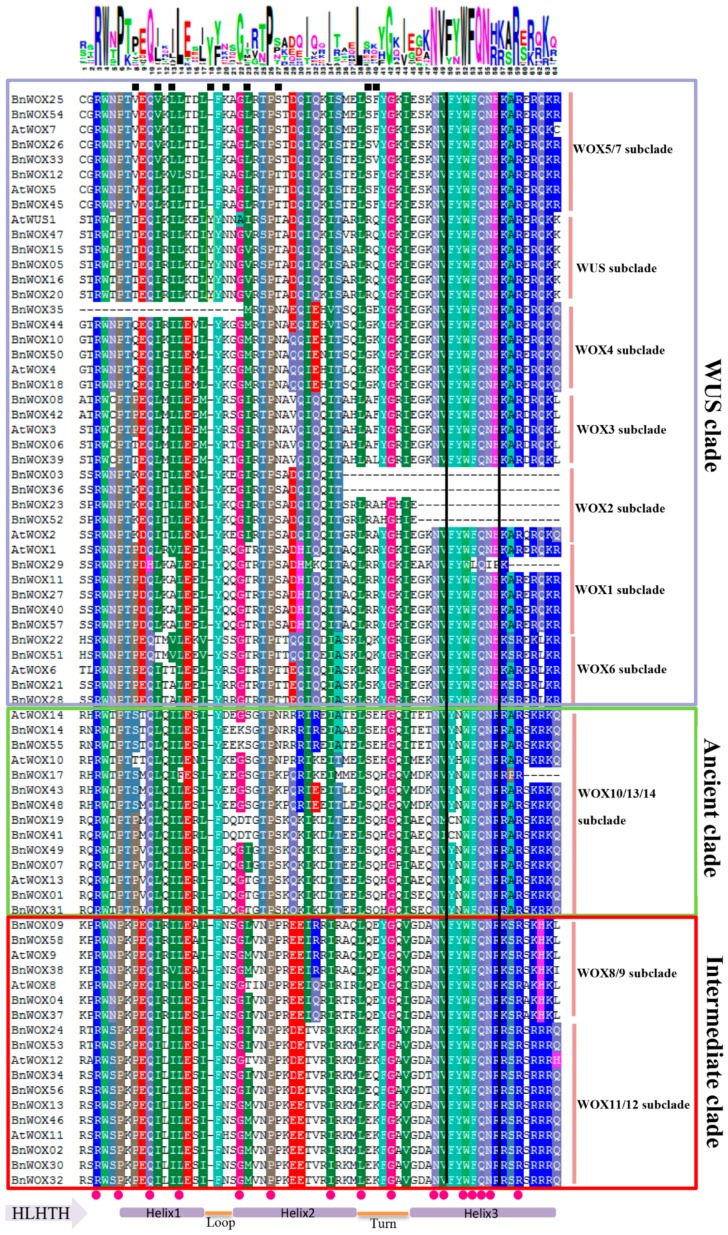
Homeodomain sequence analysis in WOX proteins from *B. napus* and *Arabidopsis*. The sequence logos are based on the alignments and the WOX family members contain the typical helix–loop–helix–turn–helix structure. Completely conserved residues are represented by red dots and WUS subclade-specific residues are indicated by black squares. Black boxes indicate marker sequences for the clades and green, red and purple boxes show the ancient, intermediate and WUS clades, respectively, with subclades. The conserved Y residue insertion at the 18th position is also indicated.

To compare the sequence features, we performed a multiple alignment analysis of the HB domains of the 58 BnWOXs using MAFFT with the default parameters [22]. The sequence logos and the secondary structures of the HB domains were generated on the Weblogo (http://weblogo.berkeley.edu/logo.cgi) and PRABI (https://npsa-prabi.ibcp.fr/cgi-bin/npsa_automat.pl?page=npsa_sopma.html) platforms (Figure 2). Our results showed that the HB domains were highly conserved and commonly contained helix–loop–helix–turn–helix structures, which were either 63 or 64 amino acid residues in length, with the exception of BnWOX23, BnWOX52, BnWOX13 and BnWOX36, which had a short amino acid deletion at the C-terminus due to incomplete genome information (Figure 2). Consistent with previous reports [3], there was a conserved Y (Tyr) residue insertion after the 17th amino acid in the HB domains of all AtWUS homologs, resulting in a total of 63 amino acids (Figure 2). In addition, 16 amino acid residues were completely conserved and mainly located in the third helix, which may play an important role in gene function [1,3]. Interestingly, there were also some conserved subclade-specific substitutions (e.g., at 8th, 37th, 20th and 23rd amino acid position) in the HB domains in the modern clade (Figure 2). However, the functional properties of these sites need to be further predicted and/or confirmed by mutational analysis, which was beyond the scope of the current study.

The FYWFQNH, FYWFQNR and YNWFQNR motifs (from 50th to 56th amino acid in the HB domain) have been reported as representative markers for the three main clades, respectively [18,23]. In the present study, we confirmed these motifs in *B. napus* as well but the Y residue in the YNWFQNR motif was replaced by C (Cys) in BnWOX19 and BnWOX41 in the ancient clade. Together, our analysis of the protein sequences indicated that the HB domains are highly conserved in BnWOXs.

### 2.4. Gene Structure and Intron Pattern Analysis

Structural diversity of genes can provide an important clue for the function and evolution of multigene families [24]; therefore, in the present study, we analyzed the gene structure of *WOX*s in *B. napus*, *B. rapa*, *B. oleracea* and *Arabidopsis* (Figure S2).

**Figure 3:**
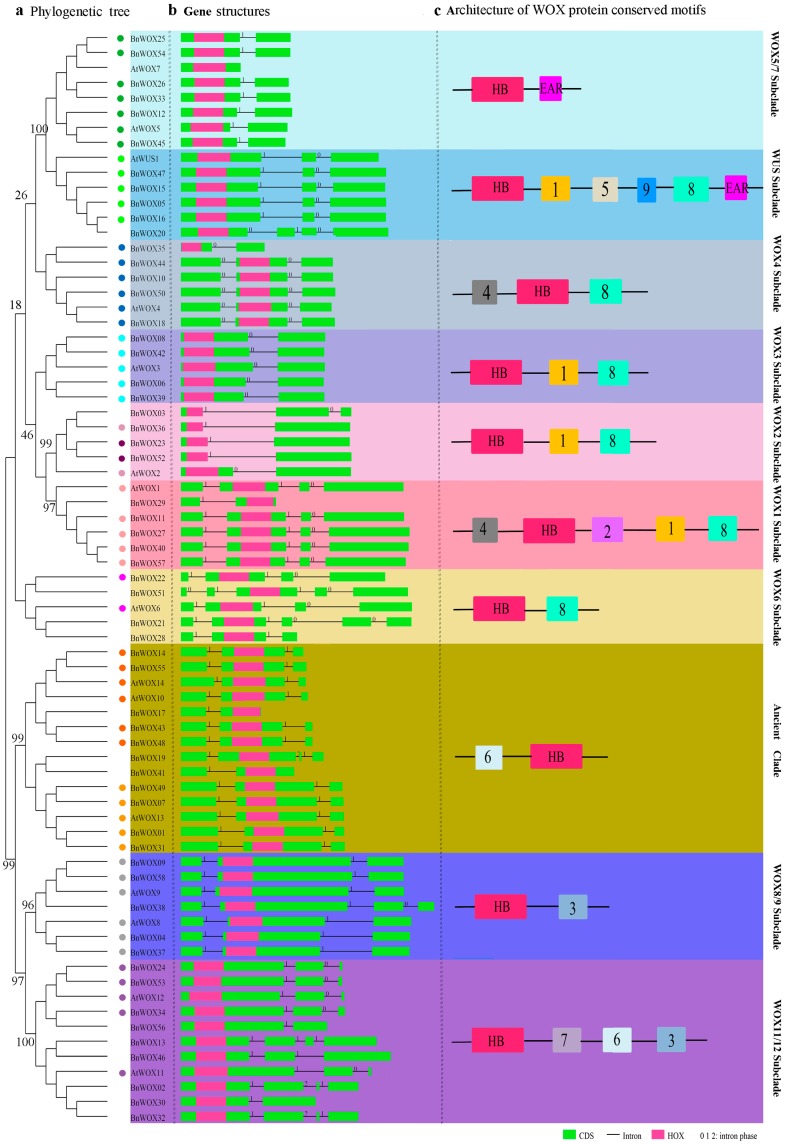
Gene structure and conserved motifs in *WOX*s from *Arabidopsis* and *B. napus*. (**a**) The neighbor-joining tree was generated from the alignment of 73 *WOX* genes from *Arabidopsis* and *B. napus*. The different background colors represent the nine subclades, which are supported by high bootstrap values. The colored dots indicate the 12 conserved intron insertion patterns. (**b**) Gene structures were generated using the Gene Structure Display Server (GSDS 2.0) [25]. Green boxes indicate exons; black lines indicate introns and pink boxes represent the HB domains. Numbers 0, 1 and 2 represent intron phases. (**c**) Conserved motifs were detected using MEME and are shown as boxes of different colors. Motif sequences are provided in Table S4.

We did not observe intron insertions in the HB domains of the 73 *WOXs* from *Arabidopsis* and *B. napus*. Members of the same subclade generally had the same or similar exon/intron patterns, except for the WOX6 and WOX11/12 subclades, which showed complex exon–intron patterns and significant variation in the number of introns present (Figure 3). The HB domains were located in the first exon in the WOX5 subclade and in the second exon in the WOX10/14 subclade, although they were commonly conserved in members of the same clade or subclade (Figure 3). Moreover, 12 conserved intron patterns were found in the *BnWOX* gene family (Table S2). Members of the ancient clade had two intron insertion sites; the first was conserved throughout all *WOX* members but the second differed between *WOX13* and *WOX10/14* homologs. In the intermediate clade, we identified three intron patterns and the intron insertion sites were similar in the WOX8/9 subclade, while members of the WOX11/12 subclade showed different intron insertion patterns and the number of introns was variable. Interestingly, the sequences at the C-terminal end of the genes in the WOX11/12 and WOX8/9 subclades were highly homologous; accordingly, several genes across these two subclades had a conserved intron insertion site in this region (Figure 3). Similarly, in the modern clade, the intron insertion sites were generally highly conserved in each subclade, except the *WOX6* subclade, which had several members with an additional intron insertion site at the C- or N-terminus. Moreover, we found that very few intron patterns were different between members of the WOX1/WOX6 and WOX5/WUS subclades because of their high homology. These results indicated that these clades or subclades may have had a common ancestor and that intron insertion might be a driving force of functional differentiation during evolution.

We predicted that 25 *BnWOX*s were complementary to 31 microRNA sequences (Table S3), which suggests that the *WOX* gene family may be regulated by microRNAs. Moreover, we found that 32%, 12% and 56% of *BnWOX*s belonged to the ancient, intermediate and modern clades, respectively, thus showing the concentration of the genes in the modern clade. This result indicated that the members of the clade might participate in a larger number of biological processes, as will be discussed below; however, the mismatch rate was as high as 33% so that the accuracy of these findings needs further experimental verification.

Overall, our results suggested that the intron loss/gain had occurred in *WOX*s outside the HB domain during the evolution of the gene family in Brassicaceae and the highly conserved or similar intron patterns that we identified further support the data of our phylogenetic analysis and classification.

### 2.5. Conserved Motif Analysis of B. napus WOX Proteins

Specific motifs are generally related to functional conversion and diversion and conserved motifs could be identified in the 58 BnWOX proteins using the MEME software and sequence alignment.

Eleven conserved motifs (motifs 1–11) were identified in the full-length BnWOX proteins (Table S4). With the exception of the conserved HB domain (motif 10) present in the members of all clades, the remaining domains (motifs) were only distributed in members of a given clade or subclade. In most cases, the same clade and subclade shared similar motif compositions, which further supported our phylogenetic analysis and classification data based on the homology of the HB domains in the WOX protein family. This indicated that some motifs were only shared by WOX proteins within the same clade or subclade, thus being subclade specific. For example, motifs 8 and 3 were present in all members of the modern and intermediate clades, respectively, while motif 7 was specific to the WOX11/12 subclade, which may be related to the acquisition of a novel function. However, several motifs were shared among clades or subclades, suggesting a common origin. For instance, motif 6 was common for the ancient clade and WOX11/12 subclade, which may indicate their close evolutionary relationship. No ancient clade-specific motif was found in this study.

In *Arabidopsis,* there are three functional domains in members of several subclades of the modern clade and these domains contribute significantly to the protein functions [3,26]. Thus, the acidic region (motif 5) can potentially function as an activator domain [2]; the WUS box (TLXLFP; motif 8) is essential for the transcriptional repressor activity and involved in the regulation of leaf blade outgrowth [5]; and the EAR-like motif (motif 11) is involved in transcriptional repression [27]. In the present study, although the acidic region and EAR-like (LXLXL) motif were not identified in the BnWOX proteins by MEME, we confirmed these two motifs in the modern clade by multi-sequence alignment analysis. The WUS box existed in most members of the modern clade, except those in the WOX7 subclade, while the acidic region was only present in the WUS subclade proteins and the EAR-like motif was merely present in the members of the WUS and WOX5/7 subclades, except AtWOX7 (Figure 3). However, the acidic region was not strictly conserved among the WUS members from *B. napus* and similar results were obtained in Solanaceae species [28], where several sites were substituted in the motif. Future research should examine whether these amino acid substitutions impact the functions of candidate genes in *B. napus*. Taken together, the organization of conserved motifs in each clade or subclade of the BnWOX gene family encoded proteins was strongly conserved. To some extent, these specific motifs may contribute to the functional divergence and conservation of BnWOX proteins.

### 2.6. Chromosomal Distribution and Duplication of B. napus WOX Genes

To investigate the relationships between genetic divergence and gene duplications within the *B. napus WOX* gene family, we determined the chromosomal location of the candidate *BnWOX*s based on the genome annotation information from the Genoscope database and visualized the data with R [29].

**Figure 4:**
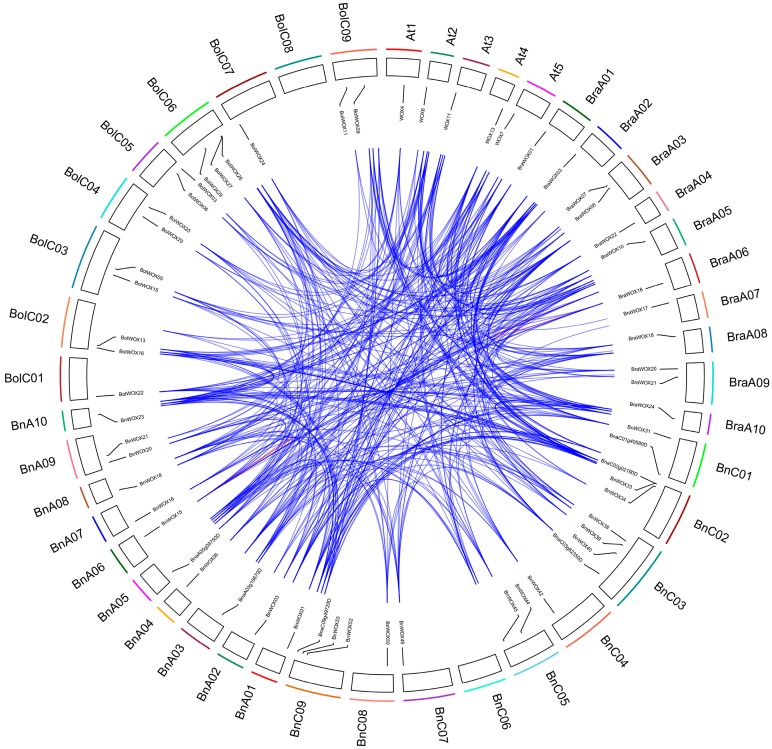
Collinearity analysis of *WOX* genes from *Arabidopsis*, *B. rapa*, *B. oleracea* and *B. napus.* The outer circle indicates the chromosome numbers and the inner circle indicates the location of *AtWOX*s, *BnWOX*s, *BrWOX*s and *BoWOX*s on the chromosomes. The blue lines link two syntenic *WOX* genes from *B. napus*, *Arabidopsis*, *B. rapa* and *B. oleracea.* The red line indicates a tandem duplication.

Our results showed that all but nine genes were located in the unanchored random regions and the last 49 *BnWOX*s were unevenly distributed on 18 of the 19 chromosomes (except for ChrC06) (Figure 4). For example, there were five genes on ChrA05 and ChrC02 each and only one on ChrA04. In general, *BnWOX*s were randomly arranged on the top and/or bottom of the chromosomes. *B. napus* (AnAnCnCn; *n* = 19) is an allotetraploid derived from the recent hybridization between *B. rapa* (AnAn; *n* = 10) and *B. oleracea* (CnCn; *n* = 9) [14] and is, therefore, an ideal species for studying the effects of polyploidy on *WOX* gene expansion. In the present study, we also identified 27 and 30 *WOX* genes in the *B. rapa* and *B. oleracea* genomes, respectively and found that these genes were similar to those in the An and Cn sub-genomes of *B. napus* [14].

Collinearity analysis revealed that there were strong orthologs among the *B. napus, B. rapa, B. oleracea* and *Arabidopsis WOX* genes (Figure 4). There was a tripling in *Brassica* species after diversion from their common ancestor with *Arabidopsis* [14]. Thus, one *Arabidopsis WOX* should theoretically correspond to three orthologs in *B. rapa* and *B. oleracea*. However, the synteny between *WOX* genes of *B. rapa* and *B. oleracea* and their *Arabidopsis* homologs was less than expected (13:27:25; Table S6), indicating that duplicated genes might have been lost during evolution [30]. As expected, 42 of the 58 *BnWOX*s (70%) found in the *B. napus* genome had a syntenic relationship, among which 32 *BnWOX*s were inherited from *BoWOX*s (18 genes) and *BrWOX*s (14 genes). These results indicate that allotetraploidy was the main force for the rapid expansion of the *WOX* gene family in *B. napus*. Moreover, eight (19%) *BnWOX*s were obtained by segmental duplication events, including two, three and three genes in the ancient, modern and intermediate clades, respectively (Table S5), while one gene was obtained by a homologous exchange. In addition, we found that eight (53%) *AtWOX*s, seven (23%) *BoWOX*s and 17 (60%) *BrWOX*s resulted from segmental duplication and whole-genome duplication in *Arabidopsis, B. oleracea and B. rapa,* respectively. Similar results have been reported for cotton [20]. We found only one putative tandem duplication, located on ChrA06 (BnaA06g25460D and BnaA06g25450D) of *B. napus*, which indicates that tandem duplications may have contributed less to the expansion of the *WOX* gene family. Overall, our results indicate that the expansion of the *WOX* gene family in the *B. napus* genome was mainly due to whole-genome duplication (polyploidy) and segmental duplication.

### 2.7. Prediction of BnWOX Protein Interaction Networks in B. napus

It has been reported that some WOX proteins function by forming complexes with other proteins [10]. However, there is still no genome-wide overview of the protein interaction network of the WOX family members. Hence, on the basis of the data publicly available in STRING [31], we predicted and constructed a protein–protein interaction network for BnWOXs, based on their orthology with AtWOXs and visualized these findings with Cytoscape [32].

First, we predicted 38 interacting protein pairs for 10 AtWOXs, based on the *Arabidopsis* data in STRING (interaction score >0.65). Among the 10 AtWOXs, one was in the intermediate clade, two in the ancient clade and seven in the modern clade, suggesting an increasing protein interaction trend in the modern clade. Among the 38 predicted proteins, 27% were peptides, 30% were TFs and the rest were other types of proteins. It is, therefore, clear that most proteins that interact with WOXs are peptides, such as CLAVATA3 (CLV3)/ESR-RELATED 40 (CLE40) and CLV3 (Table S7). CLE peptides are a well-known group of post-translationally modified signal molecules involved in cell division in SAM, the root apical meristem (RAM) and vascular meristem, which can respond to stress signals [10,33,34]. Previous reports have demonstrated that AtWUS, AtWOX4, AtWOX5 and AtWOX14 can regulate cell division in SAM, vascular meristem and RAM [10,35], respectively, while WUS–CLV interactions were shown to establish a feedback loop between stem cells and the underlying regulatory center [7]. In addition, the results of the present study showed that AtWOXs could interact with some TFs, such as GRAS, SCR and SHR (Table S7). It is interesting that homologs of WOX5, SCR and SHR have been reported to be important for root development [36]. Although their interaction was not demonstrated, the data indicated that WOX proteins might regulate the root development by interacting with GRAS proteins. 

**Figure 5:**
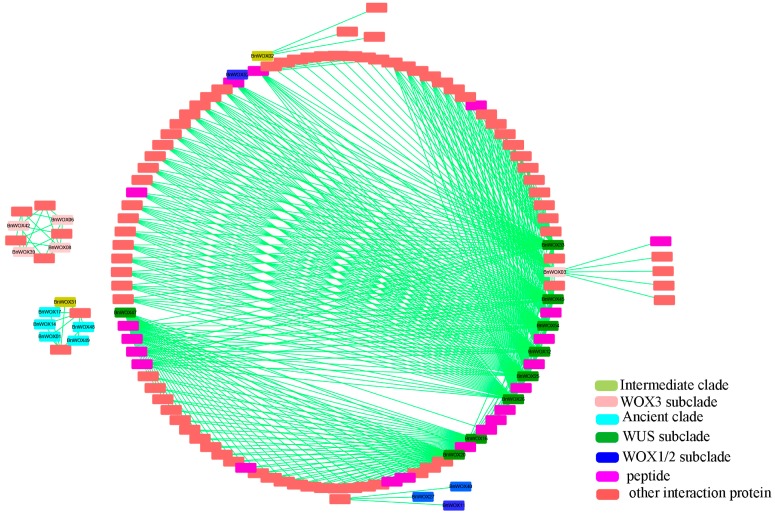
Protein interaction network of BnWOXs in *B. napus*. Different color blocks show BnWOXs from different clades and subclades, as well as different types of interacting proteins.

Based on the orthologous relationship between *B. napus* and *Arabidopsis*, a total of 463 interacting protein pairs were predicted for BnWOXs (Figure 5). Our results showed that protein interactions might be common for this gene family and that WOX proteins, especially those in the modern clade, might play a conserved role in regulating proliferation and differentiation of meristem stem cells. Our study provides important information needed to further investigate the molecular mechanisms of BnWOXs in *Brassica* species.

### 2.8. Expression Analysis of WOX Genes at Different Developmental Stages in B. napus

**Figure 6:**
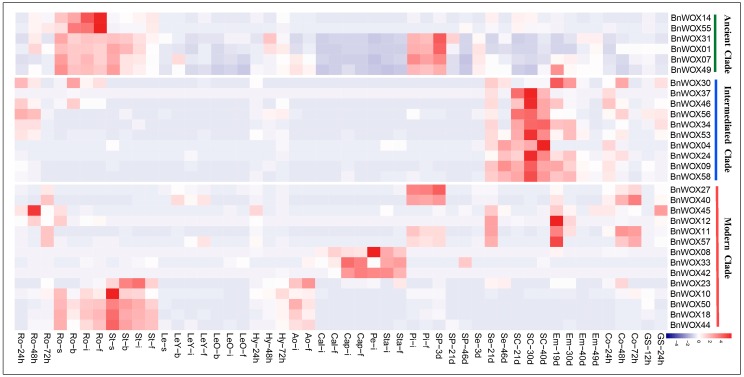
Patterns of *BnWOX* gene expression in different tissues during plant development. In the color bar at the lower right side of the figure, blue represents little or no expression, and red represents a high level of expression. Ro = root, St = stem, Le = leaf, Sp = silique pericarp, Sc = seed coat, Em = embryo, Ao = anthocaulus, Se = seed, Hy = hypocotyl, GS = germinating seed, Cap = capillament, Pi = pistil, Cal = calyx, Co = cotyledon and Pe = petal; h, d, s, b, i and f indicate hour, day, seeding, budding, initial flowering and full-bloom stages, respectively. *BnWOX*s with no or weak expression (FPKM < 1) were removed and the remaining family members were clustered by clades.

Gene expression is associated with biological function of the encoded protein; therefore, we examined the expression patterns of the 58 *BnWOX*s in 50 *B. napus* tissues/organs, based on the RNA-seq data from the Gene Expression Omnibus database at the National Center of Biotechnology Information. To make the image used for the expression analysis more intuitive, we excluded from the heatmap (Figure 6) data on 18 *BnWOX*s with no or weak expression (fragments per kilobase of transcript per million mapped reads (FPKM) < 1), which are most likely pseudogenes or expressed under certain conditions. We found that all *BnWOX*s were likely to be expressed in a limited number of vegetative tissues and reproductive organs, with relatively more genes expressed in root, stem and seed tissues, suggesting possible temporal and spatial expression patterns. Moreover, expression patterns of members of each clade were relatively conserved. We did not observe any expression of the genes from the ancient clade, except for *BnWOX19*, *BnWOX48*, *BnWOX41*, *BnWOX43* and *BnWOX13*; the remaining genes showed conserved expression patterns in roots, stems and pistils at flowering stages (Figure 6). Fourteen members of the intermediate clade were conserved and preferentially expressed in seeds, especially in the seed coat and embryo tissues, suggesting that they may contribute to seed maturation and embryonic development. This result was consistent with those of a previous report, which indicated that members of the WOX8/9 subclade regulated tissue proliferation during embryonic development in *Arabidopsis* [37]. In the modern clade, *BnWOX*s showed a relatively wider expression pattern than did those from the other two clades and were highly expressed in the roots, stems, flowers and seeds at different stages, which indicated that members of the modern clade may play tissue-specific roles. In general, the expression patterns were different for members of different subclades in the modern clade but very similar within the same subclade. For example, *BnWOX10*, *BnWOX18*, *BnWOX44* and *BnWOX50* from the WOX4 subclade were highly expressed in roots and stems, while *BnWOX27*, *BnWOX40* and *BnWOX11* from the WOX1 subclade showed high levels of expression in the pistil. The relatively wider expression patterns of members of the modern clade were consistent with the data from previous reports, which showed that this clade originated later and underwent clear functional divergence from the other clades in this gene family [2]. Together, these results show that *BnWOX*s generally have narrow expression profiles, being predominantly expressed in roots, stems and seeds and that the expression patterns in each clade or subclade reflect functional conservation.

### 2.9. Responses of BnWOX Genes to Environmental Stresses and Phytohormone Induction

Previous studies on *WOX* genes from various species have mainly focused on plant development [38,39], while their responses to environmental stresses and hormone induction were seldom evaluated. Therefore, in the present study, a comprehensive expression analysis of *BnWOX*s, based on RNA-seq data, was performed in roots after treatment with five hormones, including the auxin indole-3-acetic acid (IAA), abscisic acid (ABA), the cytokinin 6-benzyladenine (6-BA), the ethylene precursor 1-aminocyclopropane-1-carboxylic acid (ACC) and gibberellic acid (GA). Our results showed no obvious changes in the expression levels of more than half of *BnWOX*s after phytohormone treatment; however, several members of the WOX4 subclade were induced in seedling roots, which indicated their possible roles in hormone responses (Figure S3).

**Figure 7:**
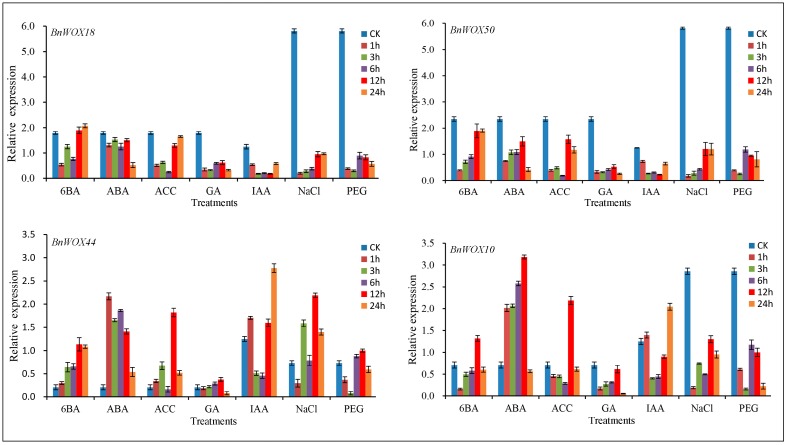
Expression levels of four *BnWOX*s after different stress treatments. Transcript levels were determined in seedling root samples by qRT-PCR under different stress treatment conditions. PEG simulated the drought stress and NaCl was used for salt stress. Gibberellic acid (GA), abscisic acid (ABA), indole-3-acetic acid (IAA), 1-aminocyclopropane-1-carboxylic acid (ACC) and 6-benzyladenine (6-BA) were used for phytohormone treatments. CK = control. Data are the mean ± standard deviation of three independent experiments.

To further elucidate these expression results and gain an insight into the expression of *BnWOX*s in response to abiotic stresses (salt and drought), four members of the WOX4 subclade (*BnWOX10*, *BnWOX50*, *BnWOX44* and *BnWOX18*) were selected to investigate their responses to hormone, salt and polyethylene glycol (PEG) stresses using qRT-PCR to evaluate changes in gene expression. The results of qRT-PCR analysis were similar to those of our RNA-seq analysis of phytohormone treatment responses, that is, the members of the WOX4 subclade were differentially regulated by different stress treatments (Figure 7). Moreover, as a sister pair, *BnWOX50* and *BnWOX18* showed similar stress responses, being repressed by almost all exogenous phytohormones, although 6-BA, which downregulated the expression of these genes at 1, 3 and 6 h, gradually reversed this inhibitory effect at 12 and 24 h. Moreover, three-fold inhibition of *BnWOX50* and *BnWOX18* expression was observed after NaCl and PEG treatments, compared with their expression in the untreated control, indicating that these genes may be involved in drought and salt resistance. On the other hand, the *BnWOX10* and *BnWOX44* genes showed differential expression patterns under some stress conditions, while being similarly upregulated by ABA treatment and downregulated after 6 h of IAA treatment. The expression of *BnWOX44* was upregulated by ACC, GA, 6-BA, NaCl and PEG treatments, while *BnWOX10* was repressed by the same treatments, indicating functional divergence between these genes. Overall, members of the WOX4 subclade may play vital roles in responses to salt and drought stresses, as well as to phytohormones, which makes these genes candidates for further study of *B. napus* abiotic stress responses.

## 3. Discussion

### 3.1. Structural and Functional Conservation of the Plant WOX Gene Family

The WOX gene family is specific to plants and has been identified in rice, sorghum, maize, *Arabidopsis* and Norway spruce [3,12,13]. *WOX* genes are well known to play key roles in the development and functional conservation in various plant species [2,6,8,40]. To confirm this conservation of the WOX gene family across land plants, we summarized the functions of *WOX* genes that have been characterized in plants to date (Table S11). We found that members of the ancient clade were mainly involved in the regulation of root development; members of the intermediate clade were involved in embryogenesis and morphological development and members of the modern clade were mainly involved in meristem maintenance. Although functional differentiation has occurred in the modern clade, the functions of members of each subclade are relatively conserved. For example, members of the WOX4 subclade in *Arabidopsis* and rice mainly promote vascularization [10]; the expression profile in *B. napus* in the present study was consistent with previous findings, further supporting functional conservation across different species.

Notably, we found that functional conservation of each clade or subclade was supported by a highly conserved gene structure, illustrated by conserved motif and intron patterns. The HB domain contains a helix–loop–helix–turn–helix structure [22], which can recognize sequence-specific targets in a precise spatial and temporal manner. Moreover, the domain is conserved in different species, thus maintaining its functional integrity [2,3]. In addition, we found representative markers, YNWFQNR, FYWFQNH and FYWFQNR, in all three clades in *B. napus*, while members of the modern and intermediate clades contained conserved substitutions at the H and R residues, which may be responsible for their functional diversification (Figure 3). Interestingly, we found that these representative markers and some conserved amino acid residues were located in the helix 3 region of the HB domain. Thus, we speculate that helix 3 plays a pivotal role in the functional differentiation between different clades. Although the functions of the proteins in the modern clade were relatively varied, they were conserved within the same subclade. There were also several conserved substitutions outside the helix 3 region in the modern clade; thus, 27%, 50%, 19% and 28% of subclade-specific sites existed in the helix 1, loop, helix 2 and turn regions, respectively, indicating that the loop and turn regions were also main regions related to the functional differentiation in the modern clade (Figure 3).

We observed that the conserved motifs outside the HB domain were specific for each clade or subclade, indicating that these motifs were likely required for specific protein functions. In the modern clade, nearly all the members (except AtWOX7) shared a conserved WUS box. It has also been reported that STF/WOX1 interacted with TOPLESS (TPL)/TPL-related proteins through the conserved WUS box to regulate the blade outgrowth by mediating cell proliferation in *Medicago truncatula* [41]. Similarly, the results of the present study revealed that TPL could interact with WOX5 homologs, suggesting that this may be a common mechanism for the repressive function of the WUS box in members of the modern clade. The EAR-like domain was shared by members of the WUS and WOX5/7 subclades and has been reported to act as a transcriptional repressor [3]. In addition, a previous report has indicated that the acidic region was related to the activation of transcription of WOX proteins [2] and was only found in the WUS subclade. These results demonstrated that specific motifs were conserved in proteins belonging to certain clades and subclades and could play important roles in the functional conservation in the WOX gene family. Consequently, the other motifs identified in this study may also be critical for gene function, although their roles still need further experimental clarification.

Introns can be specifically inserted and conserved in plant genomes and are related to functional diversity as exons are lost or gained during evolution [18]. In the present study, we found that intron insertion sites were similar or conserved in the same clades or subclades (Table S2), which further supports the division and functional conservation of subclades. Members of the WOX4 subclade, which are involved in the maintenance of vegetative and reproductive meristems [10], shared conserved patterns of introns. Differences in the intron patterns between the WOX2 and WOX1 subclades corresponded to their involvement in zygotic apical cell and lateral organ development, respectively. Thus, the intron insertion patterns may contribute to functional conservation and differentiation.

The results of our analysis of the relationships of conserved motifs and intron patterns with the gene structure and expression profiles of *BnWOX*s indicate that there are homologous genes in each clade and/or subclade, which contribute to functional conservation in different plant species. The results presented here will be useful for selecting appropriate candidate genes for further functional research in *B. napus.*

### 3.2. Conservation of Expression Profiles in Different Plants Supports Their Functional Conservation

The expression pattern of a gene often correlates with its function and previous studies have investigated the expression of *WOX*s in rice and *Arabidopsis* [3,14,42]. To further confirm the functional conservation and differentiation during the evolution of land plants, we analyzed *WOX* gene expression patterns in rice, soybean, *B. napus, B. oleracea and B. rapa* (Figure S4). Our results indicated that the expression patterns of members of the intermediate and ancient clades were generally conserved and they were expressed at high levels in green pods and roots in soybean (Figure S5). However, the expression of ancient-clade genes differed between rice and soybean, indicating that functional differentiation occurred during the evolution of monocot and dicot plants.

As mentioned above, *B. napus* is a typical allotetraploid, making it an ideal model for studying the effect of naturally occurring polyploidy on genetic conservation [14]. To investigate the functional conservation and differentiation in the *WOX* gene family, we compared the expression patterns and sequence identities/similarities of the HB domains, as well as the full-length protein, gene and promoter sequences of 32 orthologous pairs between *B. napus, B. oleracea* and *B. rapa* and of 23 sister pairs from *B. napus* (Table S9). We found that *BoWOX*s and *BrWOX*s were also mainly expressed in vegetative (root, stem and leaf) and reproductive (flower and silique) organs. Most members of the intermediate clade showed no or very low expression levels compared to those of members of the other two clades, which may indicate that the former are pseudogenes or need to be induced by specific stresses. The expression of *WOX*s from the ancient and intermediate clades was conserved across *B. napus, B. oleracea* and *B. rapa* and was mainly observed in roots (Figure S3). Our results also showed that most sister pairs shared a very high degree of sequence identity, with 96.9% identity in the HB domains and 86.32% identity in the full-length proteins. Accordingly, 73% (17 of 23) of the *BnWOX* paralogs shared similar expression patterns. Among these sister pairs, 80%, 85% and 55% belonged to the ancient, intermediate and modern clades, respectively, implying that these genes are functionally redundant. For example, the *BnWOX09* and *BnWOX58* pair from the intermediate clade was highly expressed in seed tissues, while the *BnWOX55* and *BnWOX14* pair from the ancient clade was highly expressed in roots. In contrast, several sister pairs underwent neo-functionalization or sub-functionalization, especially in the modern clade, so that their expression patterns were slightly or obviously different. For instance, in the *BnWOX24* and *BnWOX53* pair, only *BnWOX53* was highly expressed in the root (Figure 6). Furthermore, the promoter regions of sister pairs were significantly homologous, with the average sequence identity ranging from 20% to 86% (Table S9). In addition, the expression patterns of sister pairs were closely related to their promoter sequence identities. Thus, we found that several sister pair genes that were differentially expressed had high sequence identity in the HB regions but less identity in the promoter regions, suggesting that the functional divergence of homologous *WOX* genes may have first occurred in the promoter regions during their evolution.

In this study, we identified 18 and 14 orthologous pairs from the *WOX* gene family in *B. rapa* and *B. oleracea* using the identity in the HB domain, which was 93.6% and 89.9%, respectively. However, the expression patterns of 62% of the orthologous pairs were slightly divergent or significantly different. For example, *BnWOX53* and *BoWOX12* were both expressed in roots but *BnWOX53* was also expressed in seeds, indicating that functional divergence occurred during the evolution of these two genes. Interestingly, approximately 60%, 20% and 20% of the *WOX* genes that exhibited differential expression belonged to the modern, intermediate and ancient clades, respectively (Table S8), further supporting the hypothesis that the modern clade emerged recently in land plants and underwent functional diversification during the evolution. Furthermore, the average levels of identity of the promoter regions of orthologous pairs ranged from 8% to 99.7% and were generally much lower than those of the ORF regions, indicating that the promoter regions play vital roles in the functional diversity of *WOX* genes. Taken together, comparison of the expression patterns of *WOX* genes across different land plants revealed the major functional conservation and/or slight differentiation during the evolution of this gene family and indicated that the functional differentiation of these genes may be related to sequence diversity in their promoter regions.

## 4. Materials and Methods

### 4.1. Identification of WOX Proteins and Phylogenetic Analysis of the B. napus Genome

The sequences of 15 *Arabidopsis WOX* genes were downloaded from the TAIR *Arabidopsis* genome (http://www.arabidopsis.org/) [43] to aid in the identification of candidate genes encoding WOX proteins in *B. napus*. We performed a BLASTP search of the Genoscope genome database (http://www.genoscope.cns.fr/brassicanapus/) using the DNA-binding domain protein sequences of the *Arabidopsis WOX* genes as queries. To verify the reliability of our results, the protein functional and structural domains were predicted by PROSITE profiling (http://www.expasy.org/tools/scanprosite/) [44] to confirm that each protein had the HB domain. We acquired the *B. oleracea* and *B. rapa* WOX protein sequences from Phytozome (https://phytozome.jgi.doe.gov/pz/portal.html) and BRAD (http://brassicadb.org/brad/index.php) using the same method as that used for *Arabidopsis*.

The biochemical properties of the candidates were predicted using ExPASy [45] and subcellular localization was investigated using Cell-PLoc [46].

To gain insights into the evolutionary history of the *WOX* gene family in *B. napus*, *B. rapa*, *B. oleracea* and *Arabidopsis*, we constructed an NJ tree based on a multiple sequence alignment of the HB domains, using MEGA version 7.0 [16] with the following parameters: Poisson correction, pairwise deletion and a bootstrap with 1000 replicates. Tree files were viewed and edited with FigTree v1.3.1 (http://tree.bio.ed.ac.uk/software/figtree/). The MEGA 7.0 program was used to estimate the best model for the multiple sequence alignment of the HB domains by default. The JJT amino acid substitution model with estimation of the gamma distribution shape parameter (JJT + G + I) was suggested to be the best evolutionary models, based on the Akaike information criterion (AIC)statistics. An ML tree was constructed using PhyML 3.0 [17] with 100 replicates and the JJT + G + I model.

### 4.2. Sequence, Gene Structure and Conserved Motif Analysis and Construction of the Protein Interaction Network

To analyze the sequence features of the BnWOXs proteins, we performed a multiple alignment analysis of the HB domains using MAFFT version 7 with the default parameters (https://mafft.cbrc.jp/alignment/server/). To obtain optimized alignments, the deduced amino acid sequences were adjusted manually in MEGA 7.0 and BioEdit 7.0 (http://www.psc.edu/biomed/genedoc/) [16] with the default parameters. The intron patterns, including the distribution, position and phases and the HB domain positions in the *B. napus WOX* genes were analyzed using the GSDS software 2.0 (http://gsds.cbi.pku.edu.cn/) [25]. The intron insertion information for the *WOX* genes of *Arabidopsis*, *B. rapa* and *B. oleracea* was acquired from Phytozome (https://phytozome.jgi.doe.gov/pz/portal.html).

The MEME version 4.11.1 [47] program was used to identify potential protein motifs, in addition to the HB domain, using the following parameter settings for the distribution of motifs: the maximum number of motifs, 10; the minimum width of a motif, 6; and the maximum width of a motif, 50. Only motifs with an E-value ≤ 1 × 10^−10^ were used for further analysis. The interaction network was constructed based on the orthologs of *BnWOX*s in *Arabidopsis* using the STRING platform (https://string-db.org/?tdsourcetag=s_pctim_aiomsg) and visualized with Cytoscape version 3.4.0 (Chongqing, China).

### 4.3. Chromosomal Location and Synteny Analysis

The gene loci for *B. napus WOX* genes were extracted from the Genoscope genome database. The synteny relationships of *WOX* genes from *Arabidopsis*, *B. napus*, *B. oleracea* and *B. rapa* were acquired from CoGe (https://genomevolution.org/coge/). The R package [29] was used to view the chromosomal locations of the candidates and their collinearity.

### 4.4. Analysis of Expression Profiles of *BnWOX*s at Different Developmental Stages and After Hormone and Stress Treatment

The temporal and spatial expression patterns of candidate *BnWOX*s were further analyzed using the RNA-seq data from 50 different tissues, which included roots, stems, leaves, flowers, seeds and siliques from the *B. napus* cultivar ”Zhongshuang 11” (ZS11) at different developmental stages (e.g., germination, seedling, budding, initial flowering and full-bloom stages). Seedling roots treated with hormones (e.g., IAA, GA, 6-BA, ABA and ACC) were also collected for analysis. The data were analyzed with Cluster 3.0 (Chongqing, China) [32] and a heatmap was drawn using the R package.

For qRT-PCR, seeds of ZS11 were obtained from the College of Agriculture and Biotechnology, Southwest University (Chongqing, China) and germinated on Petri dishes. At the five-leaf stage, seedlings were treated with Hoagland’s liquid medium, which contained a 15% (*w*/*v*) PEG 6000 solution to simulate a drought condition, 200 mM NaCl, or phytohormones (50 µM ABA, 120 µM GA, 75 µM 6-BA, 60 µM ACC and 10 µM IAA), and grown in an artificial climate chamber at 25 °C under a 14-h/10-h (day/night) photoperiod. Root tissue was harvested at 0, 1, 3, 6, 12 and 24 h of treatment, immediately frozen in liquid nitrogen and stored at −80 °C for RNA isolation.

Extraction of total RNA from root samples and subsequent cDNA synthesis were performed as described previously [48]. The SYBR Premix ExTaq™ II kit (Takara Biotechnology, Dalian, China) was used for qRT-PCR amplification in a CFX Connect™ real-time PCR system (Bio-Rad, Chongqing, China) and the SYBR Green PrimeScript RT-PCR kit (Takara Biotechnology). Each reaction was conducted in a 10 µL volume and contained 5 µL of PCR master mix, 2.5 µL of double distilled H_2_O (ddH_2_O), 2 µL of diluted template and 0.25 µL of each gene-specific primer (Table S10). Three biological replicates were performed and three technical replicates were taken for each biological replicate. The *Actin7* gene (GenBank accession no. AF024716) was used as an internal control. The reaction conditions for real-time PCR were as follows: initial denaturation at 95 °C for 3 min, followed by 40 cycles of denaturation at 95 °C for 10 s and annealing at 58 °C for 30 s. The relative gene expression levels were calculated using the 2^−ΔΔ*C*t^ method [49].

## Figures and Tables

**Table 1:** Features of the 58 *BnWOX* genes from *Brassica napus*, identified in this study.

Gene	Genome ID	Chromosome	Protein Length (Amino Acids)	cDNA Length (bp)	Introns/Exons	pI	Molecular Weight (Da)	Subcellular Localization
*BnWOX01*	BnaA01g01910D	chrA01	256	940	2/3	5.72	28,458.4	Nucleus
*BnWOX02*	BnaA01g34040D	chrA01	289	870	3/4	6.48	31,778.6	Nucleus
*BnWOX03*	BnaA02g07100D	chrA02	175	528	2/3	6.36	19,236.2	Nucleus
*BnWOX04*	BnaA02g24210D	chrA02	320	1053	2/3	6.96	35,591.2	Nucleus
*BnWOX05*	BnaA02g36550D	chrA02	302	909	2/3	7.09	34,348.7	Nucleus
*BnWOX06*	BnaA03g22070D	chrA03	226	681	1/2	9.33	26,087	Nucleus
*BnWOX07*	BnaA03g53400D	chrA03	263	1036	2/3	5.3	29,170.3	Nucleus
*BnWOX08*	BnaA04g16520D	chrA04	234	705	1/2	9.32	27,143.2	Nucleus
*BnWOX09*	BnaA05g09770D	chrA05	390	1173	2/3	8.35	43,341.9	Nucleus
*BnWOX10*	BnaA05g18600D	chrA05	251	756	2/3	9.63	28,555.1	Nucleus
*BnWOX11*	BnaA05g22250D	chrA05	351	1287	3/4	8.59	40,110.9	Nucleus
*BnWOX12*	BnaA05g27750D	chrA05	191	718	1/2	8.87	22,361	Nucleus
*BnWOX13*	BnaA05g32800D	chrA05	326	981	3/4	6.63	35,661	Nucleus
*BnWOX14*	BnaA06g14590D	chrA06	203	768	2/3	5.43	22,850.2	Nucleus
*BnWOX15*	BnaA06g25450D	chrA06	294	885	2/3	7.81	33,475.7	Nucleus
*BnWOX16*	BnaA07g02390D	chrA07	302	909	2/3	6.85	34,305.6	Nucleus
*BnWOX17*	BnaA07g11310D	chrA07	133	402	1/2	7.89	15,260.3	Nucleus
*BnWOX18*	BnaA08g04100D	chrA08	256	942	2/3	9.31	29,143.6	Nucleus
*BnWOX19*	BnaA08g14960D	chrA08	239	720	3/4	5.73	27,383.7	Nucleus
*BnWOX20*	BnaA09g09400D	chrA09	316	1022	3/4	6.89	35,862	Nucleus
*BnWOX21*	BnaA09g18650D	chrA09	267	804	4/5	6.41	30,920.3	Nucleus
*BnWOX22*	BnaA09g45340D	chrA09	275	901	3/4	6.28	31,962	Nucleus
*BnWOX23*	BnaA10g12360D	chrA10	210	633	1/2	6.83	23,085.3	Nucleus
*BnWOX24*	BnaA10g16970D	chrA10	274	949	2/3	6.34	30,342.5	Nucleus
*BnWOX25*	BnaA10g24790D	chrA10	192	579	1/2	9.17	22,114.9	Nucleus
*BnWOX26*	BnaAnng01480D	chrAnn_random	191	576	1/2	6.66	21,832.3	Nucleus
*BnWOX27*	BnaAnng11050D	chrAnn_random	353	1062	3/4	6.97	40,197.6	Nucleus
*BnWOX28*	BnaAnng35720D	chrAnn_random	166	501	2/3	8.99	19,173.4	Nucleus
*BnWOX29*	BnaAnng36010D	chrAnn_random	121	366	1/2	9.61	13,962.8	Nucleus
*BnWOX30*	BnaAnng41310D	chrAnn_random	247	847	1/2	8.77	27,574.8	Nucleus
*BnWOX31*	BnaC01g03050D	chrC01	265	1016	2/3	5.67	29,250.4	Nucleus
*BnWOX32*	BnaC01g40590D	chrC01	289	972	3/4	6.48	31,778.6	Nucleus
*BnWOX33*	BnaC02g02200D	chrC02	191	576	1/2	5.18	46,938.7	Nucleus
*BnWOX34*	BnaC02g07490D	chrC02	283	852	2/3	5.08	71,825.3	Nucleus
*BnWOX35*	BnaC02g08530D	chrC02	121	366	1/2	6.09	31,334.6	Nucleus
*BnWOX36*	BnaC02g10040D	chrC02	199	600	1/2	5.95	21,671.7	Nucleus
*BnWOX37*	BnaC02g32050D	chrC02	320	1277	2/3	6.96	35,542.2	Nucleus
*BnWOX38*	BnaC03g18850D	chrC03	397	1303	3/4	6.57	44,157.5	Nucleus
*BnWOX39*	BnaC03g26450D	chrC03	222	669	1/2	9.26	25,657.6	Nucleus
*BnWOX40*	BnaC03g40380D	chrC03	350	1053	3/4	7.72	40,024.6	Nucleus
*BnWOX41*	BnaC03g62360D	chrC03	171	641	1/2	7.65	19,697.3	Nucleus
*BnWOX42*	BnaC04g39860D	chrC04	236	711	1/2	9.3	27,260.4	Nucleus
*BnWOX43*	BnaC04g46990D	chrC04	195	588	2/3	5.51	21,785.3	Nucleus
*BnWOX44*	BnaC05g25380D	chrC05	251	908	2/3	9.58	28,526	Nucleus
*BnWOX45*	BnaC05g41930D	chrC05	191	718	1/2	8.87	22,485.1	Nucleus
*BnWOX46*	BnaC05g48100D	chrC05	339	1020	3/4	5.98	37,085.8	Nucleus
*BnWOX47*	BnaC07g06960D	chrC07	302	2162	2/3	7.36	34,407.9	Nucleus
*BnWOX48*	BnaC07g15200D	chrC07	195	588	2/3	5.35	21,686.1	Nucleus
*BnWOX49*	BnaC07g45680D	chrC07	261	1022	2/3	5.23	29,076.2	Nucleus
*BnWOX50*	BnaC08g04810D	chrC08	255	938	2/3	9.43	28,912.4	Nucleus
*BnWOX51*	BnaC08g39150D	chrC08	290	873	4/5	5.93	33,988.5	Nucleus
*BnWOX52*	BnaC09g34650D	chrC09	210	633	1/2	6.58	23,144.4	Nucleus
*BnWOX53*	BnaC09g40100D	chrC09	276	1794	2/3	5.97	30,547.8	Nucleus
*BnWOX54*	BnaC09g49730D	chrC09	192	579	1/2	8.99	22,099.8	Nucleus
*BnWOX55*	BnaCnng49040D	chrCnn_random	209	579	2/3	5.48	23,619.1	Nucleus
*BnWOX56*	BnaCnng49570D	chrCnn_random	280	843	1/2	5.97	31,047.2	Nucleus
*BnWOX57*	BnaCnng51820D	chrCnn_random	351	1314	3/4	8.4	40,001.6	Nucleus
*BnWOX58*	BnaCnng62530D	chrCnn_random	390	1954	2/3	8.35	43,232.7	Nucleus

## Supplementary Materials

Supplementary materials can be found at http://www.mdpi.com/1422-0067/19/11/3470/s1.

## Abbreviations

ABA	abscisic acid
ACC	1-aminocyclopropane-1-carboxylic acid
CLV3	CLAVATA3
CLE40	CLAVATA3/ESR-RELATED 40
FPKM	fragments per kilobase of transcript per million mapped reads
GA	gibberellic acid
GSDS	gene structure display server
HB domain	homeodomain
IAA	indole-3-acetic acid
ORF	open reading frame
PEG	polyethylene glycol
qRT-PCR	quantitative real-time polymerase chain reaction
RAM	root apical meristem
RNA-seq	RNA sequencing
SAM	shoot apical meristem
TPL	TOPLESS
WOX	WUSCHEL-related homeobox
ZS11	”Zhongshuang 11”

## References

[1] Mayer K.F., Schoof H., Haecker A., Lenhard M., Jürgens G., Laux T. (1998). Role of WUSCHEL in regulating stem cell fate in the *Arabidopsis* shoot meristem. Cell.

[2] Vandenbussche M., Horstman A., Zethof J., Koes R., Rijpkema A.S., Gerats T. (2009). Differential recruitment of WOX transcription factors for lateral development and organ fusion in petunia and *Arabidopsis*. Plant Cell.

[3] Zhang X., Zong J., Liu J., Yin J., Zhang D. (2010). Genome-wide analysis of WOX gene family in rice, sorghum, maize, *Arabidopsis* and poplar. J. Integr. Plant Biol..

[4] Haecker A., Gross-Hardt R., Geiges B., Sarkar A., Breuninger H., Herrmann M., Laux T. (2004). Expression dynamics of WOX genes mark cell fate decisions during early embryonic patterning in *Arabidopsis thaliana*. Development.

[5] Lin H., Niu L., McHale N.A., Ohme-Takagi M., Mysore K.S., Tadege M. (2013). Evolutionarily conserved repressive activity of WOX proteins mediates leaf blade outgrowth and floral organ development in plants. Plant Signal. Behav..

[6] Sarkar A.K., Luijten M., Miyashima S., Lenhard M., Hashimoto T., Nakajima K., Scheres B., Heidstra R., Laux T., Sarkar A.K. (2007). Conserved factors regulate signalling in *Arabidopsis thaliana* shoot and root stem cell organizers. Nature.

[7] Schoof H., Lenhard M., Haecker A., Mayer K.F., Jürgens G., Laux T. (2000). The stem cell population of *Arabidopsis* shoot meristems in maintained by a regulatory loop between the CLAVATA and WUSCHEL genes. Cell.

[8] Breuninger H., Rikirsch E., Hermann M., Ueda M., Laux T. (2008). Differential expression of WOX genes mediates apical-basal axis formation in the *Arabidopsis* embryo. Dev. Cell.

[9] Wu X., Dabi T., Weigel D. (2005). Requirement of homeobox gene STIMPY/WOX9 for *Arabidopsis* meristem growth and maintenance. Curr. Biol..

[10] Etchells J.P., Provost C.M., Mishra L., Turner S.R. (2013). WOX4 and WOX14 act downstream of the PXY receptor kinase to regulate plant vascular proliferation independently of any role in vascular organisation. Development.

[11] Kamiya N., Nagasaki H., Morikami A., Sato Y., Matsuoka M. (2003). Isolation and characterization of a rice WUSCHEL-type homeobox gene that is specifically expressed in the central cells of a quiescent center in the root apical meristem. Plant J..

[12] Nardmann J., Werr W. (2006). The shoot stem cell niche in angiosperms: Expression patterns of WUS orthologues in rice and maize imply major modifications in the course of mono- and dicot evolution. Mol. Biol. Evol..

[13] Zhu T., Moschou P.N., Alvarez J.M., Sohlberg J.J., Arnold S.V. (2014). WUSCHEL-RELATED HOMEOBOX 8/9 is important for proper embryo patterning in the gymnosperm *Norway spruce*. J. Exp. Bot..

[14] Chalhoub B., Denoeud F., Liu S., Parkin I.A., Tang H., Wang X., Chiquet J., Belcram H., Tong C., Samans B. (2014). Early allopolyploid evolution in the post-Neolithic *Brassica napus* oilseed genome. Science.

[15] He Z., Cheng F., Li Y., Wang X., Parkin I.A., Chalhoub B., Liu S., Bancroft I. (2015). Construction of *Brassica* A and C genome-based ordered pan-transcriptomes for use in rapeseed genomic research. Data Brief.

[16] Kumar S., Stecher G., Tamura K. (2016). MEGA7: Molecular evolutionary genetics analysis version 7.0 for bigger datasets. Mol. Biol. Evol..

[17] Stéphane G., Frédéric D., Jean-François D., Olivier G. (2009). Estimating maximum likelihood phylogenies with PhyML. Methods Mol. Biol..

[18] Cao Y., Han Y., Meng D., Li G., Li D., Abdullah M., Jin Q., Lin Y., Cai Y. (2017). Genome-wide analysis suggests the relaxed purifying selection affect the evolution of WOX genes in *Pyrus bretschneideri*, *Prunus persica*, *Prunus mume*, and *Fragaria vesca*. Front. Genet..

[19] Nardmann J., Werr W. (2013). Symplesiomorphies in the WUSCHEL clade suggest that the last common ancestor of seed plants contained at least four independent stem cell niches. New Phytol..

[20] Yang Z., Gong Q., Qin W., Yang Z., Cheng Y., Lu L., Ge X., Zhang C., Wu Z., Li F. (2017). Genome-wide analysis of WOX genes in upland cotton and their expression pattern under different stresses. BMC Plant Biol..

[21] Lian G., Ding Z., Wang Q., Zhang D., Xu J. (2014). Origins and Evolution of WUSCHEL-related homeobox protein family in plant kingdom. Sci. World J..

[22] Katoh K., Standley D.M. (2013). MAFFT multiple sequence alignment software version 7: Improvements in performance and usability. Mol. Biol. Evol..

[23] Zeng M., Hu B., Li J., Zhang G., Ying R., Huang H., Wang H., Xu L. (2016). Stem cell lineage in body layer specialization and vascular patterning of rice root and leaf. Sci. Bull..

[24] Hedman H., Zhu T., Arnold S.V., Sohlberg J.J. (2013). Analysis of the WUSCHEL-RELATED HOMEOBOX gene family in the conifer *picea abies* reveals extensive conservation as well as dynamic patterns. BMC Plant Biol..

[25] Hu B., Jin J., Guo A.Y., Zhang H., Luo J., Gao G. (2014). GSDS 2.0: An upgraded gene feature visualization server. Bioinformatics.

[26] Ikeda M., Ohme-Takagi M. (2009). *Arabidopsis* WUSCHEL is a bifunctional transcription factor that acts as a repressor in stem cell regulation and as an activator in floral patterning. Plant Cell.

[27] Paponov I.A., Teale W., Lang D., Paponov M., Reski R., Rensing S.A., Palme K. (2009). The evolution of nuclear auxin signalling. BMC Evol. Biol..

[28] Li X., Hamyat M., Liu C., Salman A., Gao X., Guo C., Wang Y., Guo Y. (2018). Identification and characterization of the WOX family genes in five *Solanaceae Species* reveal their conserved roles in peptide signaling. Genes.

[29] Zhang H., Meltzer P., Davis S. (2013). RCircos: An R package for Circos 2D track plots. BMC Bioinform..

[30] Lynch M., Conery J.S. (2000). The evolutionary fate and consequences of duplicate genes. Science.

[31] Szklarczyk D., Morris J.H., Cook H., Kuhn M., Wyder S., Simonovic M., Santos A., Doncheva N.T., Roth A., Bork P. (2017). The STRING database in 2017: Quality-controlled protein–protein association networks, made broadly accessible. Nucleic acids Res..

[32] Shannon P., Markiel A., Ozier O., Baliga N.S., Wang J.T., Ramage D., Amin N., Schwikowski B., Ideker T. (2003). Cytoscape: A software environment for integrated models of biomolecular interaction networks. Genome Res..

[33] Fletcher J.C., Brand U., Running M.P., Simon R., Meyerowitz E.M. (1999). Signaling of cell fate decisions by CLAVATA3 in *Arabidopsis* shoot meristems. Science.

[34] Matsubayashi Y. (2014). posttranslationally modified small-peptide signals in plants. Annu. Rev. Plant Biol..

[35] Lin T.F., Saiga S., Abe M., Laux T. (2016). OBE3 and WUS interaction in shoot meristem stem cell regulation. PLoS ONE.

[36] Della R.F., Fattorini L., D’Angeli S., Veloccia A., Del D.S., Cai G., Falasca G., Altamura M.M. (2015). *Arabidopsis* SHR and SCR transcription factors and AUX1 auxin influx carrier control the switch between adventitious rooting and xylogenesis in planta and in vitro cultured thin cell layers. Ann. Bot..

[37] Wu X., Chory J., Weigel D. (2007). Combinations of WOX activities regulate tissue proliferation during *Arabidopsis* embryonic development. Dev. Biol..

[38] Cheng S., Huang Y., Zhu N., Zhao Y. (2014). The rice WUSCHEL-related homeobox genes are involved in reproductive organ development, hormone signaling and abiotic stress response. Gene.

[39] Liu J., Sheng L., Xu Y., Li J., Yang Z., Huang H., Xu L. (2014). WOX11 and 12 are involved in the first-step cell fate transition during de novo root organogenesis in *Arabidopsis*. Plant Cell.

[40] Dolzblasz A., Nardmann J., Clerici E., Causier B., van der Graaff E., Chen J., Davies B., Werr W., Laux T. (2016). Stem cell regulation by *Arabidopsis* WOX genes. Mol. Plant.

[41] Zhang F., Wang Y., Li G., Tang Y., Kramer E.M., Tadege M. (2014). STENOFOLIA recruits TOPLESS to repress ASYMMETRIC LEAVES2 at the leaf margin and promote leaf blade outgrowth in *Medicago truncatula*. Plant Cell.

[42] Zhao Y., Hu Y., Dai M., Huang L., Zhou D.X. (2009). The WUSCHEL-related homeobox gene WOX11 is required to activate shoot-borne crown root development in rice. Plant Cell.

[43] Berardini T.Z., Reiser L., Li D., Mezheritsky Y., Muller R., Strait E., Huala E. (2015). The *Arabidopsis* information resource: Making and mining the "gold standard" annotated reference *Plant Genome*. Genesis.

[44] Thompson J.D., Gibson T.J., Plewniak F., Jeanmougin F., Higgins D.G. (1997). The CLUSTAL_X windows interface: Flexible strategies for multiple sequence alignment aided by quality analysis tools. Nucleic acids Res..

[45] Artimo P., Jonnalagedda M., Arnold K., Baratin D., Csardi G., de Castro E., Duvaud S., Flegel V., Fortier A., Gasteiger E. (2012). ExPASy: SIB bioinformatics resource portal. Nucleic Acids Res..

[46] Chou K.C., Shen H.B. (2007). Cell-PLoc: A package of Web servers for predicting subcellular localization of proteins in various organisms. Nat. Protoc..

[47] Bailey T.L., Boden M., Buske F.A., Frith M., Grant C.E., Clementi L., Ren J., Li W.W., Noble W.S. (2009). MEME SUITE: Tools for motif discovery and searching. Nucleic Acids Res..

[48] Zhang J., Zhang S., Li H., Du H., Huang H., Li Y., Hu Y., Liu H., Liu Y., Yu G. (2016). Identification of transcription factors ZmMYB111 and ZmMYB148 involved in phenylpropanoid metabolism. Front. Plant Sci..

[49] Livak K.J., Schmittgen T.D. (2001). Analysis of relative gene expression data using real-time quantitative PCR and the 2^−ΔΔ*C*t^ method. Methods.

