# Supplementary material for: Global Analysis of WOX Transcription Factor Gene Family in Brassica napus Reveals Their Stress- and Hormone-Responsive Patterns
Source: Int J Mol Sci. 2018 Nov 5;19(11):3470. doi: 10.3390/ijms19113470 (PMC6274733; doi:10.3390/ijms19113470)
Supplement: Supplementary file 1 [file ijms-19-03470-s001.zip › ijms-372054-SI/Supplementary Figures.pdf]

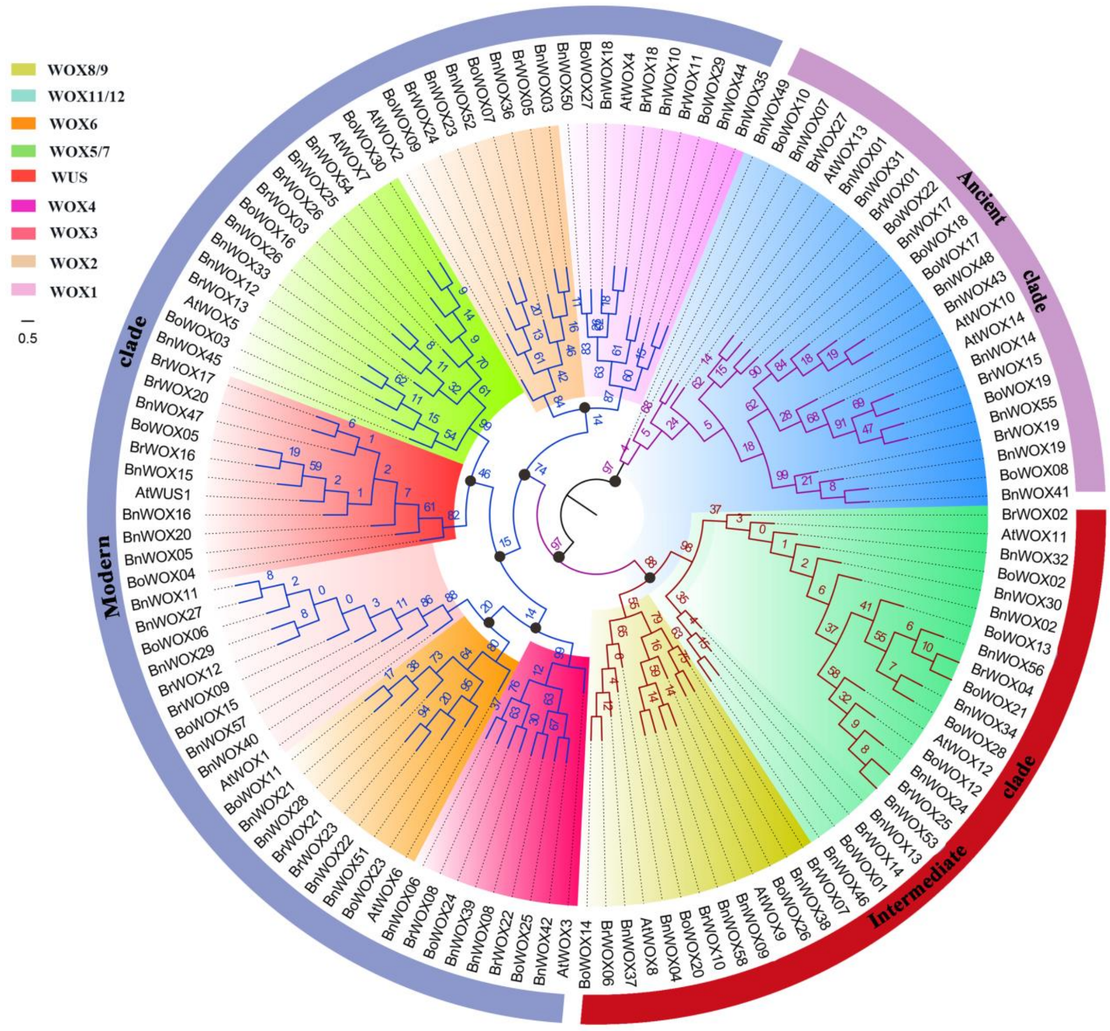

Figure S1. Maximum Likelihood (ML) phylogenetic tree of 130 *WOX* genes from *Arabidopsis* (At), *B. rapa* (Br), *B. oleracea* (Bo), and *B. napus* (Bn). The phylogenetic tree was generated based on the alignment of 130 WOX homeodomain sequences from *Arabidopsis* (15 genes), *B. rapa* (27 genes), *B. oleracea* (30 genes), and *B. napus* (58 genes) with 100 bootstrap replicates by PhyML3.0. The outer circle represents the three clades, is marked in purple, blue, and brown colors. Nine sub-clades were classified and are marked in different background colors; background and the bootstrap values are shown near the nodes.

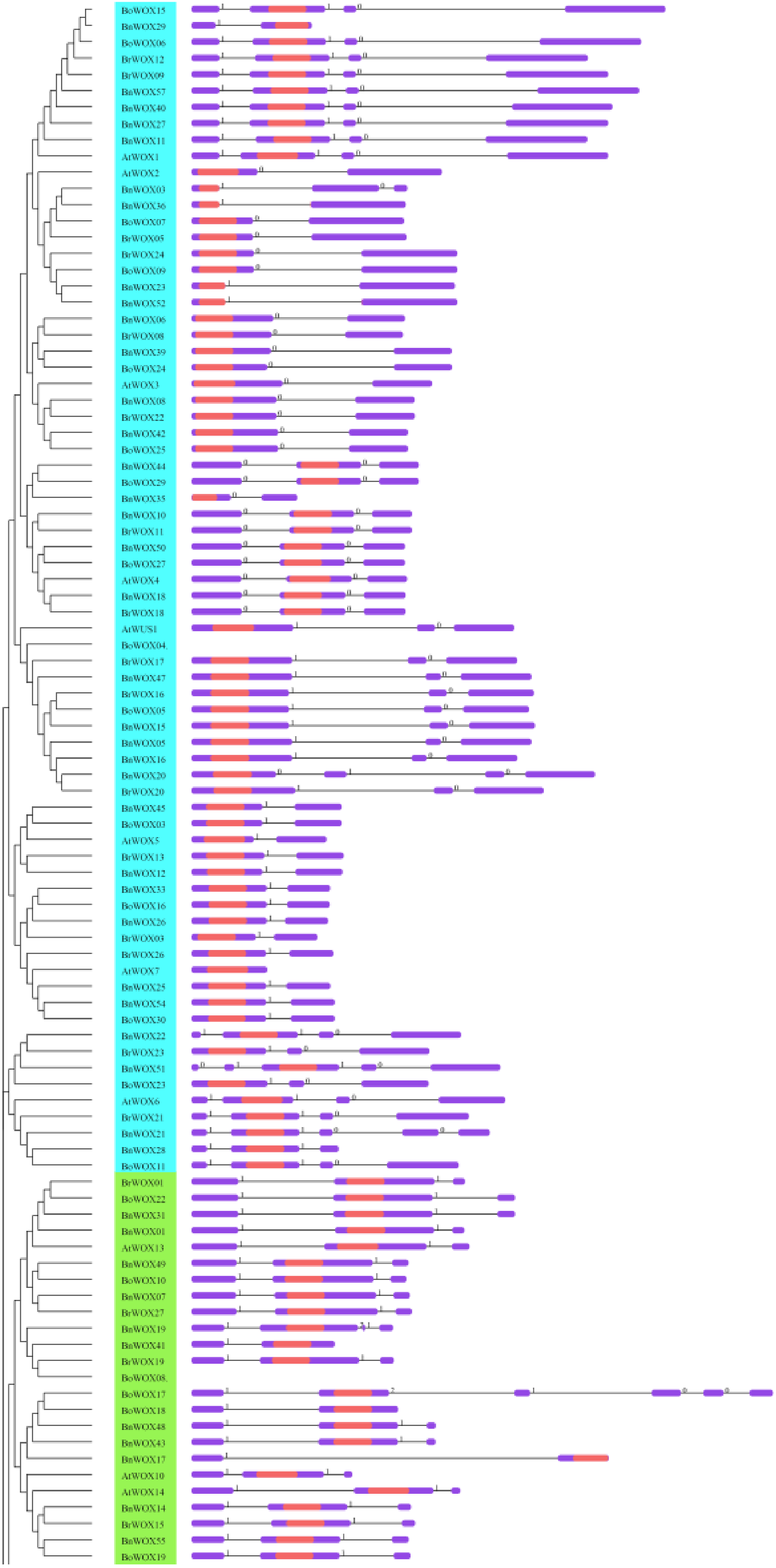

BoWOX15
BnWOX29
BoWOX06
BrWOX12
BrWOX09
BnWOX57
BnWOX40
BnWOX27
BnWOX11
AtWOX1
AtWOX2
BnWOX03
BnWOX36
BoWOX07
BrWOX05
BrWOX24
BoWOX09
BnWOX23
BnWOX52
BnWOX06
BrWOX08
BnWOX39
BoWOX24
AtWOX3
BnWOX08
BrWOX22
BnWOX42
BoWOX25
BnWOX44
BoWOX29
BnWOX35
BnWOX10
BrWOX11
BnWOX50
BoWOX27
AtWOX4
BnWOX18
BrWOX18
AtWUS1
BoWOX04
BrWOX17
BnWOX47
BrWOX16
BoWOX05
BrWOX15
BnWOX05
BnWOX16
BnWOX20
BrWOX20
BnWOX45
BoWOX03
AtWOX5
BrWOX13
BnWOX12
BnWOX33
BoWOX16
BnWOX26
BrWOX03
BrWOX26
AtWOX7
BnWOX25
BnWOX54
BoWOX30
BnWOX22
BrWOX23
BnWOX51
BoWOX23
AtWOX6
BrWOX21
BnWOX21
BnWOX28
BoWOX11
BrWOX01
BoWOX22
BnWOX31
BnWOX01
AtWOX13
BnWOX49
BoWOX10
BnWOX07
BrWOX27
BnWOX19
BnWOX41
BrWOX19
BoWOX08
BoWOX17
BoWOX18
BnWOX48
BnWOX43
BnWOX17
AtWOX10
AtWOX14
BnWOX14
BrWOX15
BnWOX55
BoWOX19

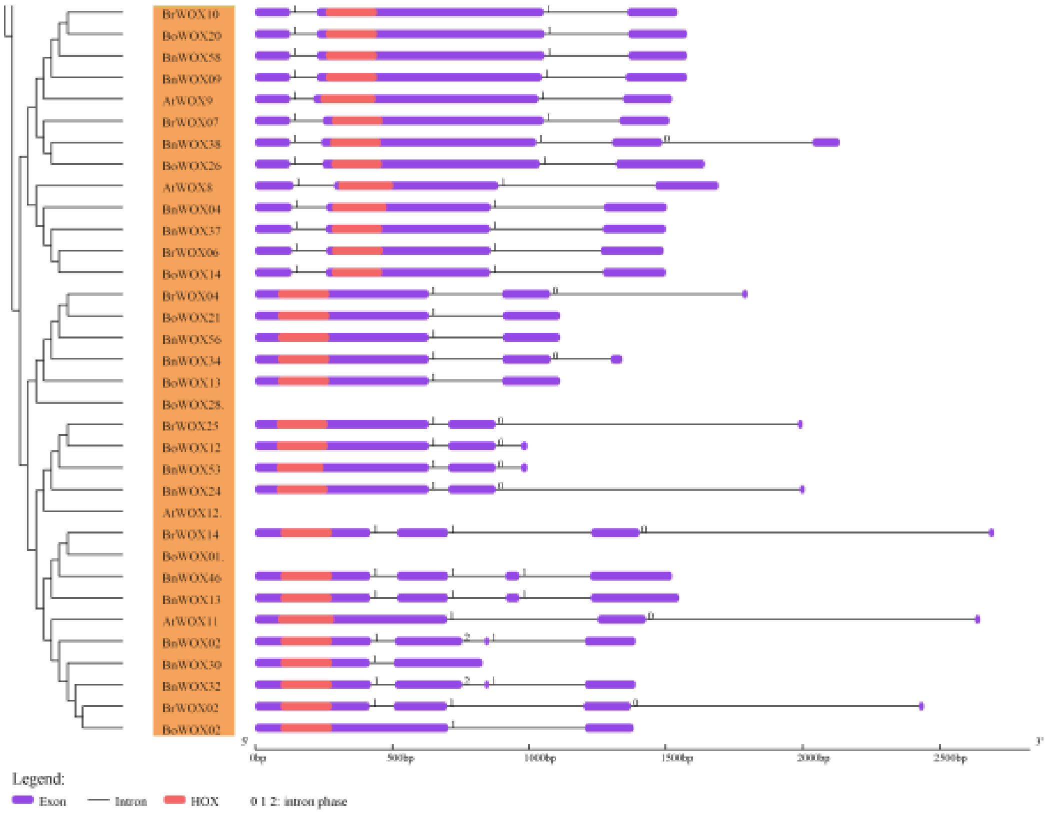

Figure S2. Phylogenetic tree and gene structures of 130 *WOX* genes. The blue, green and orange background showed the three clades (ancient, intermediate and modern clades) respevtively. The structures of WOX genes from *B. napus*, *Arabidopsis*, *B. olerace* and *B. rape* were shown by GSDS software respectively. Purple boxes indicate the exons, black lines indicate the introns, and orange boxes represent the HB domains. Numbers 0, 1and 2 represent introns in phases 0, 1, and 2.

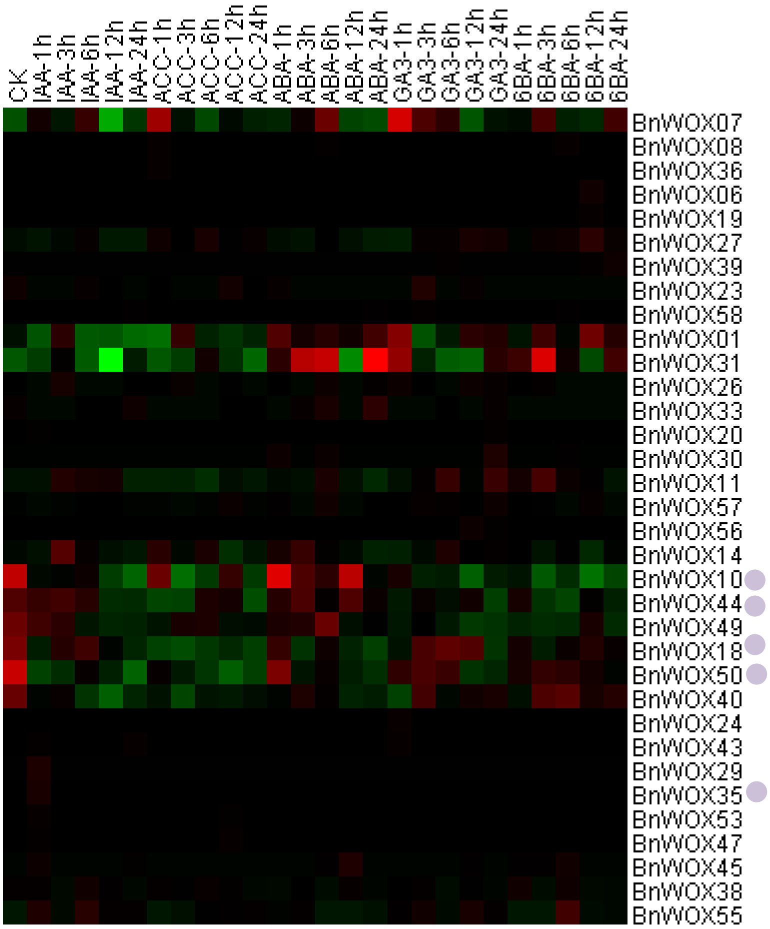

Figure S3. Expression profile of *BnWOX* genes under hormone treatments. *BnWOX* genes were treated under five hormones (GA: Gibberellin, ABA: abscisic acid, IAA: Auxin, ACC: Ethylene, 6BA: Cytokinin), the purple dots represent the members of WOX4 subclade.

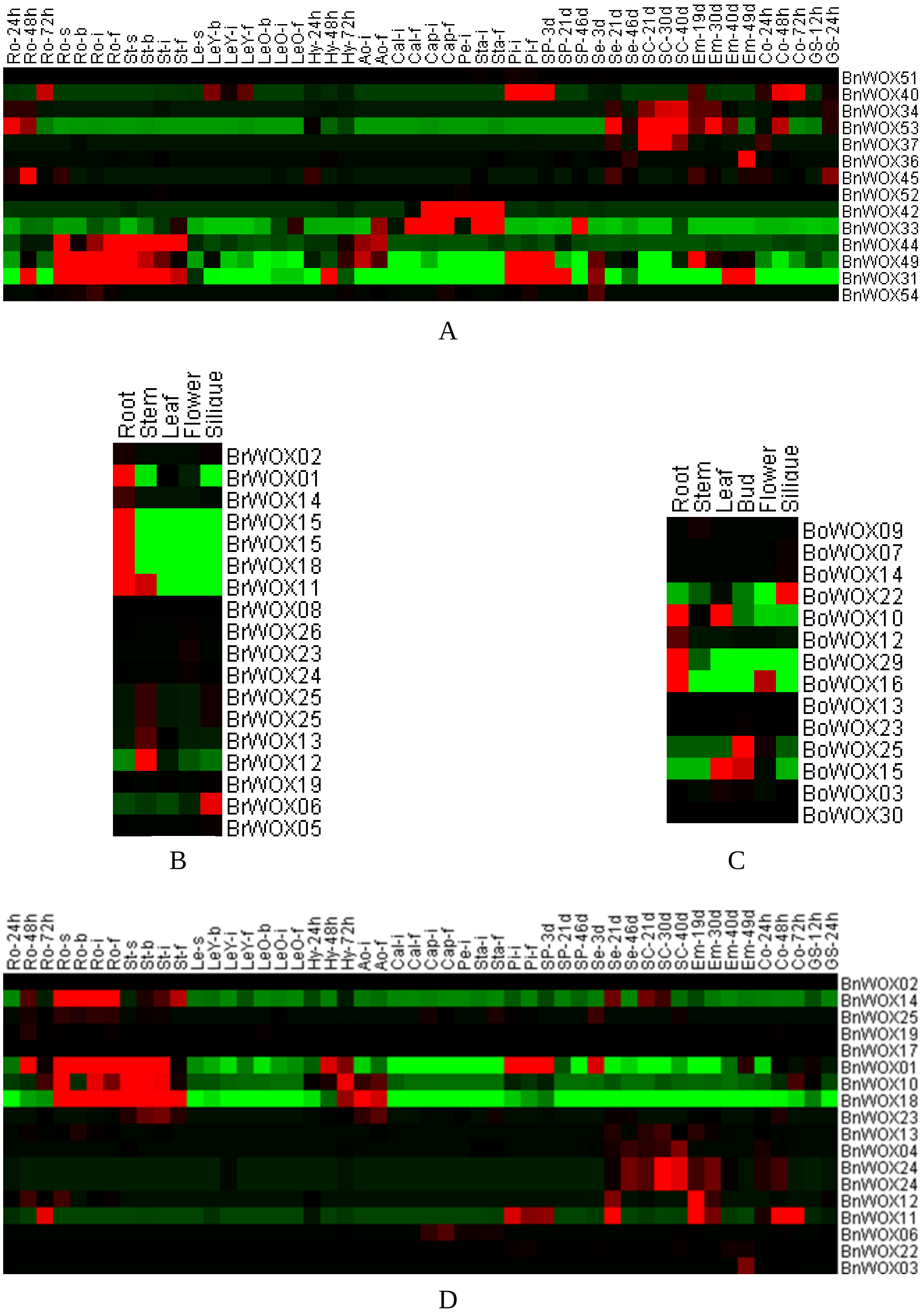

Figure S4. Expression profile of *BnWOXs* orthologous pairs from *B. rapa* and *B. oleracea*. A and B indicate the expression patterns of WOX orthologous pairs across *B. napus* (*BnWOXs*) and *B. rapa* (*BoWOXs*). C and D indicate the expression pattern of WOX orthologous pairs across *B.napus* (*BnWOXs*) and *B. rapa* (*BrWOXs*). Expression patterns of *BnWOX* genes were represented in different tissues during different development. Ro = root, St = stem, Le = leaf, Sp = silique pericarp, Sc = seed coat, Em = embryo, Ao = anthocaulus, Se = seed, Hy = hypocotyl, GS =germination seeds, Cap = capillament, Pi = pistil, Cal = calyx, Co = cotyledon, Pe = petal. The "h", "d", "i", "f", "s" indicate hour, day, seeding, budding, initial flowering, and full-bloom stages, respectively.

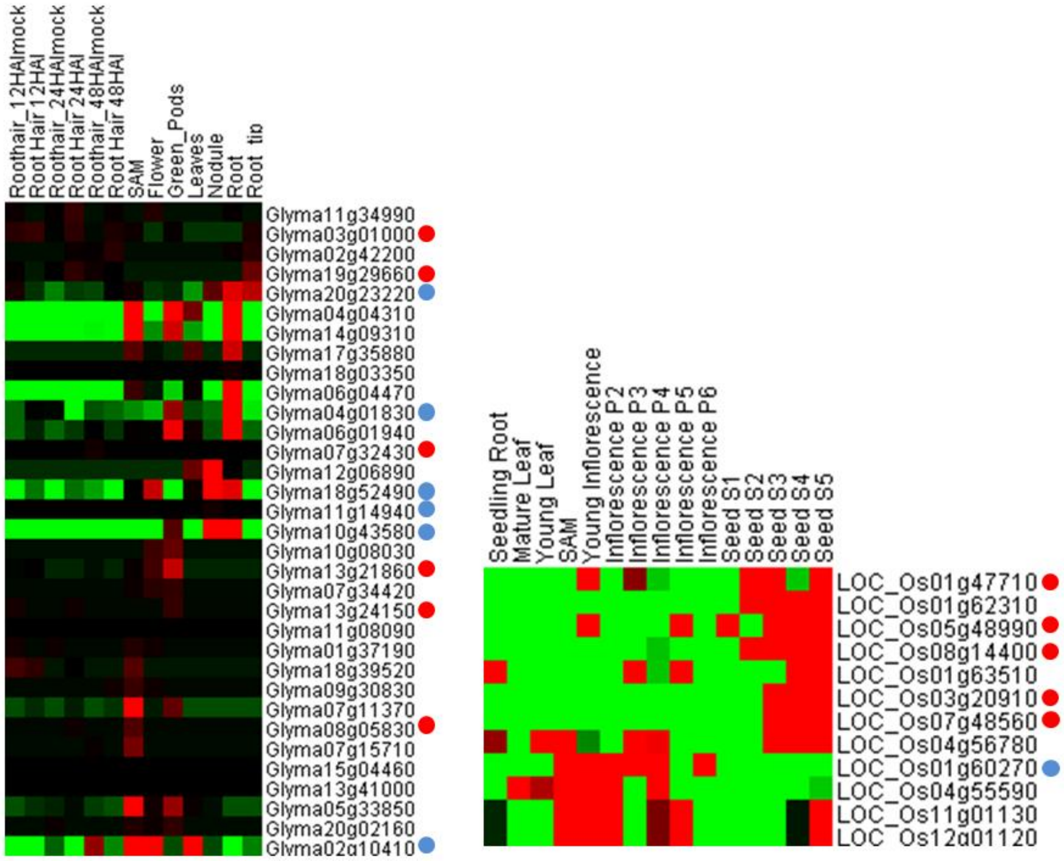

A B

Figure S5. Expression patterns of *WOX* genes in different tissues in *Glycine max* and *Oryza sativa*. A: The expressions of *WOX* genes in *Glycine max*; B: The expression of *WOX* genes in *Oryza sativa.* The blue and red dots represent the members of ancient and intermediate clades, the last represent members of modern clade.
