# Supplementary material for: Global Analysis of WOX Transcription Factor Gene Family in Brassica napus Reveals Their Stress- and Hormone-Responsive Patterns
Source: Int J Mol Sci. 2018 Nov 5;19(11):3470. doi: 10.3390/ijms19113470 (PMC6274733; doi:10.3390/ijms19113470)
Supplement: Supplementary file 1 [file ijms-19-03470-s001.zip › ijms-372054-SI/Table S10.pdf]

Table S10. List of the primers used for the Real-time PCR analyses

Genome ID.	Name	Forward primer (5'-3')	Reverse primer (5'-3')	Target size (bp)
BnaC05g25380D	*BnWOX44*	CAAGAGTCGCCATCATCCC	GATCGAAAGTAGCGGAAGTAACA	118
BnaA05g18600D	*BnWOX10*	GAACCCAACTCAAGAACAGATAAA	CGTATTTACCGAGTTGAGAAGTTAT	110
BnaC08g04810D	*BnWOX50*	CTTCATCAGCTTAAGTTGCCT	AGTCTCTCCTGATCACGTCAT	107
BnaA08g04100D	*BnWOX18*	GACAACAACAACATCGTCATT	CTACATGTCCTCTTGTACTCAGTT	96
BnaC02g00690D	*Actin-7*	GGTTCGACCATGTTCCCAGGT	GTGCTGAGGGATGCAAGGATG	149
