# Supplementary material for: Global Analysis of WOX Transcription Factor Gene Family in Brassica napus Reveals Their Stress- and Hormone-Responsive Patterns
Source: Int J Mol Sci. 2018 Nov 5;19(11):3470. doi: 10.3390/ijms19113470 (PMC6274733; doi:10.3390/ijms19113470)
Supplement: Supplementary file 1 [file ijms-19-03470-s001.zip › ijms-372054-SI/Table S11.pdf]

Table S11. The characterized functions of plant *WOX* genes

Clade	WOX homologs	Expression tissues/organ	Functions	Species	Reference
Modern	*WUS*	SAM, ovules, anther, leaf primordium	maintaining the stem cell and population; regulating zygote development	*Arabidopsis, P. abies, P. Mulberry*	[1] [2]
	WOX1	lateral organ primordial, leaf margin	regulating flower and lateral organ development	*Arabidopsis, P. sativum L.*	[3]
	WOX2	early embryo, cotyledon, and zygote	regulating zygotic apical cell development and morphogenesis of the early embryo	*Arabidopsis, P. abies, N. spruce*	[4][5]
	WOX3	peripheral area of SAM, leaf and floral organ primordial, lateral sepals and stipules	promoting cell division forming horizontal regions of vegetative, floral organs ,lateral sepals and stipules	*Arabidopsi,s* rice	[6][7]
	WOX4	vascular, cambial meristem	Promoting vascularization and differentiation; involving in the maintenance of vegetative and reproductive meristem	*Arabidopsis*, rice	[8]
	WOX5	shoot, root meristem , vascular cambium, QC and leaf marginal meristem	maintaining stem cells of root apical meristem(RAM) and root columella stem cells	*P. Mulberry*, *Arabidopsis*, rice, wheat	[8] [2]
	WOX6	organ primordium, ovule	supressing organ primordium differentiation, regulating flower and ovule development	*Arabidopsis*	[9]
	WOX7	lateral root	inhibiting lateral root formation	*Arabidopsis, P. Mulberry*	[2][10]
Intermediate	*WOX8*	embryo, cotyledon, zygote basal cells	regulating patterning and morphogenesis of the early embryo	*Arabidopsis*, *P. abies*	[8]
	WOX9	embryo, zygote, apical cells root, SAM, seeds	maintaining cell division and inhibiting SAM differentiation ,regulating zygote development and morphogenesis of the early embryo	*Arabidopsis*	[8]
	WOX11	cambium, adventitious roots, seeds	involving in crown root growth and development; de novo root organogenesis and callus formation	*Arabidopsis, P. tomentosa,* rice	[9][12]
Ancient	*WOX12*	root , primary, xylem, seeds	promoting de novo root organogenesis and callus formation	*Arabidopsis*, rice	[13]
	WOX13	primary root, lateral root flower, SAM and RAM	promoting primary lateral root initiation, development and the floral transition; reprogramming protoplast cells into stem cells	*Arabidopsis, P. patens, C. lanceolata*	[14]
	WOX14	anther early stages of lateral root formation	affecting primary lateral root initiation and the floral transition regulation vascularization and differentiation	*Arabidopsis*	[14]

Reference:

1. Hedman, H.; Zhu, T.; Arnold, S.V.; Sohlberg, J.J. Analysis of the WUSCHEL-RELATED HOMEOBOX gene family in the *conifer picea abies* reveals extensive conservation as well as dynamic patterns. *BMC Plant Biology* **2013**, 13, 89-89. DOI: 10.1186/1471-2229-13-89. PMID: 23758772
2. Tang, F.; Chen, N.; Zhao, M.; Wang, Y.; He, R.; Peng, X.; Shen, S. Identification and Functional Divergence Analysis of WOX Gene Family in *Paper Mulberry*. *International Journal of Molecular Sciences* **2017**, 18, 1782. DOI: 10.3390/ijms18081782. PMID: 28813005
3. Li, L.; Zhuang; Mike; Ambrose; Catherine; Rameau; Weng; Yang; Xiao, H. LATHYROIDES, Encoding a WUSCHEL-Related Homeoboxl Transcription Factor, Controls Organ Lateral Growth, and Regulates Tendril and Dorsal Petal Identities in *Garden Pea* (Pisum sativum L.). *Molecular Plant*. **2012**, 1333-1345. DOI: 10.1093/mp/sss067. PMID: 22888154
4. Cheng, S.; Huang, Y.; Zhu, N.; Zhao, Y. The rice WUSCHEL-related homeobox genes are involved in reproductive organ development, hormone signaling and abiotic stress response. *Gene* **2014**, 549, 266-274. DOI: 10.1016/j.gene.2014.08.003. PMID: 25106855
5. Palovaara, J.H.H.; Stasolla, C.; Hakman, I. Comparative expression pattern analysis of WUSCHEL-related homeobox 2 (WOX2) and WOX8/9 in developing seeds and somatic embryos of the gymnosperm *Picea abies. New Phytologist* **2010**, 188, 122-135. DOI: 10.1111/j.1469-8137.2010.03336.x. PMID: 20561212
6. Sung-Hwan Cho; Soo-Cheul Yoo; Haitao Zhang; Pandeya, D.; Koh, H.J.; Hwang, J.Y.; Kim, G.T.; Paek, N.C. The rice narrow leaf2 and narrow leaf3 loci encode WUSCHEL-related homeobox 3A (OsWOX3A) and function in leaf, spikelet, tiller and lateral root development. *New Phytologist* **2013**, 198, 1071-1084. DOI: 10.1111/nph.12231. PMID: 23551229
7. Shimizu, R.; Ji, J.; Kelsey, E.; Ohtsu, K.; Schnable, P.S.; Scanlon, M.J. Tissue specificity and evolution of meristematic WOX3 function. *Plant Physiology* **2009**, 149, 841. DOI: 10.1104/pp.108.130765. PMID: 19073779
8. Etchells, J.P.; Provost, C.M.; Mishra, L.; Turner, S.R. WOX4 and WOX14 act downstream of the PXY receptor kinase to regulate plant vascular proliferation independently of any role in vascular organisation. *Development* 2013, 140, 2224-2234. DOI: 10.1242/dev.091314. PMID: 23578929
9. Deveaux, Y.; Toffano-Nioche, C.; Claisse, G.; Thareau, V.; Morin, H.; Laufs, P.; Moreau, H.; Kreis, M.; Lecharny, A. Genes of the most conserved WOX clade in plants affect root and flower development in *Arabidopsis*. *BMC Evolutionary Biology* **2008**, 8, 291. DOI: 10.1186/1471-2148-8-291 PMID: 18950478
10. Danyu; Kong; Yueling; Hongchang. The WUSCHEL Related Homeobox Protein WOX7 Regulates the Sugar Response of Lateral Root Development in Arabidopsis thaliana. *Molecular Plant* **2016**, 9, 261-270. DOI: 10.1016/j.molp.2015.11.006 . PMID: 26621542
11. Breuninger, H.; Rikirsch, E.; Hermann, M.; Ueda, M.; Laux, T. Differential expression of WOX genes mediates apical-basal axis formation in the Arabidopsis embryo. *Developmental cell* **2008**, 14, 867-876,. DOI:10.1016/j.devcel.2008.03.008. PMID: 18539115
12. Cheng, S.; Tan, F.; Lu, Y.; Liu, X.; Li, T.; Yuan, W.; Zhao, Y.; Zhou, D.X. WOX11 recruits a histone H3K27me3 demethylase to promote gene expression during shoot development in rice. *Nucleic acids research* **2018,** 46. DOI: 10.1093/nar/gky017. PMID: 29361035
13. Liu, J.; Sheng, L.; Xu, Y.; Li, J.; Yang, Z.; Huang, H.; Xu, L. WOX11 and 12 are involved in the first-step cell fate transition during de novo root organogenesis in *Arabidopsis*. *Plant Cell* **2014**, 26, 1081-1093. DOI: 10.1105/tpc.114.122887. PMID: 24642937
14. Denis, E.; Kbiri, N.; Mary, V.; Claisse, G.; Natalia, C.E.S.; Kreis, M.; Deveaux, Y. WOX14 promotes bioactive gibberellin synthesis and vascular cell differentiation in *Arabidopsis*. *Plant Journal* **2017**, 90. DOI: 10.1111/tpj.13513. PMID: 28218997
