# Supplementary material for: Global Analysis of WOX Transcription Factor Gene Family in Brassica napus Reveals Their Stress- and Hormone-Responsive Patterns
Source: Int J Mol Sci. 2018 Nov 5;19(11):3470. doi: 10.3390/ijms19113470 (PMC6274733; doi:10.3390/ijms19113470)
Supplement: Supplementary file 1 [file ijms-19-03470-s001.zip › ijms-372054-SI/Table S2.pdf]

Table S2. The twelve classified intron patterns within the WOX gene family in *B. napus*

I	MWTMGYNEGGADSFNGGRKLRPLIPRLTSCPTAAVNTNSDHRFN[1]**M**AVVTMTAEQNKRELMMLNSEPQ<br>HPPVMVSSRWNPTPDQLRVLEELYRQGTRTPSADHIQQITAQLRRYGKIEGKNVFYWFQNHKARERQK<br>RRRQMETGHEETVLSTASLVSNHGFDKKDPP[1]**G**YKVEQVKNWICSVGCDTQPE[0]**K**PSRDYHLEEPANIRV<br>EHNARCGGDERRSFLGINTTWQMMQLPPSFYSSSHHHHQRNLILNSPTVSSNMSNSNNAVSASKDTVT<br>VSPVFLRTREATNTETCHRNGDDNKDQEQHEDCSNGELDHQEQTLELFPLRKEGFCSDGEKDKNISGIH<br>CFYEFLPLKN
II	MENEVNAGTASSSRWNPTKDQITLLENLYKEGIRTPSADQIQQITGRLRAYGHIEGKNVFYWFQNHKAR<br>QRQKQKQERMAYFNRLLHKTSRFFYPPPCSN[0]**V**GCVSPYYLQQASDHHMNQHGSVYTNDLLHRNNVM<br>IPSGGYEKRTVTQHQKQLSDIRTTAATRMPISPSSLRFDRFALRDNCYAGEDINVNSSGRKTLPLFPLQPL<br>NASNADGMGSSSFALGSDSPVDCSSDGAGREQPFIDFFSGGSTSTRFDSNGNGL
III	MENEGSAGTGSSPRWNPTKEQITLLENLYKQGIRTPSADQIQQITSRLRAHGHIE[1]**V**GCVNPYYLHQVRD<br>HHNQHGSVYRQDLHSNKVMLPSGGYEERTTTDHKKHVSDITTTTARMSMSSSSLRFDRFALGNHGYFS<br>EGINVNTNGLKTLPLFPLQPLDAANDSGVGNSSFSLRHDSPVTYCGDDGGRGQPFIGFFSSGSSRFDNSG<br>NGL
IV	MSPVASTRWCPTPEQLMILEEMYRSGIRTPNAVQIQQITAHLAFYGRIEGKNVFYWFQNHKARDRQKLR<br>KKLAKQLHQQQHQLQLQLQQIKPKPISSMISQPVNKNIIDHHNPYHHHHHNHHHNHHRPYDHMSFDC<br>CSHPSPMCLPHQ[0]**G**TGVGEAPSKVMNEYYCTKSGAEEILMQKSITGPNSSYGRDWMMMMDMGPRPSY<br>PSSSSSPISCCNMMMSSPKIPLKTLELFPISSINSKQDSTKL
V	MKVHEFSNGFSSSWDQHDSTSSLSLSCKRLRPLAPKLSGSPPSPPSSSSGVTSATFDLKNFIRPDQTGPTK<br>FEHKRDPPH[0]**Q**LETHPGGTRWNPTQEQIGILEMLYKGGMRTPNAQQIEHHTLQLGKYGKIEGKNVFYWF<br>QNHKARERQKQKRNNLISLSCQSSFTTTGVFNPSVTMKTRTSSSLDIM[0]**R**EPMVEKEELVEENEYKRTCR<br>SWGFENLEIENRRNKNSSTMATTFNKIIDNVTLELFPLHPEGR
VI	MEPPQHQHHHHQADQESGNNNNNKSGSGGYTCRQTSTRWTPTTEQIKILKELYYNNAIRSPTADQIQKI<br>TARLRQFGKIEGKNVFYWFQNHKARERQKKRFNGTNMTTPSSSPNSVMMAANDHYHPLLHH[illegible]HGVP<br>MQRPANSVNVKLNQDHHLYHHNKPYPSFNN[1]**G**NLNHASSGTECGVVNASNGYMSSHVYGSM[1]**E**QDCS<br>MNYNNVGGGWANMDHHYSSAPYNFFDRAKPLFGLEGHQEEEECGGDAYLEHRRTLPLFPMHGEDHI<br>NGGSGAIWKYGQSEVRPCASLELRLN
VII	MSFSVKGRSLRGNNNGGTGTKCGRWNPTVEQLKILTDLFRAGLRTPTTDQIQKISTELSFYGKIESKNVF<br>YWFQNHKARERQKRRKISIDFDHHHHQPSTR[1]**D**VFEISEEDCQEEEKVIETLQLFPVNSFEDSNSKVDKM<br>RARGNNQYREYIRETTTTSFSPYSSCGAEMEHPPPLDLRLSFL
VIII	MGYISNNNLINYLPLSTTQPPLLLTH[1]**C**DINGNDHHQLITASSGEHDIDERKNNIPAAATLRWNPTPEQITT<br>LEELYRSGTRTPTTEQIQQIASKLRKYGRIEGKNVFYWFQNHKARERLKRRRREGGAIIKPHKDVKDSS[1]**S**<br>GGHRVDQTKLCPSFPHTNRPQP[1]**Q**HELDPASYNKDNNANNEDHGTTEESDQRASEVGKYATWRNLVTW<br>SITQQPEEINIDENVNGEEEETRDNRTLNLFPVREYQEKTGRLIEKTKACNYCYYYEFMPLKN
IX	MMEWDNQLQPNNHHSSNLQGIDVNGGSGAGGGMYVKVMTDEQYETLRKQIAIYGTICERLVEMHKT<br>LTAQQDLA[1]**G**GRMGGLYADPMMSSLGHKMTARQRWTPTPVQLQILERIFDQGTGTPSKQKIKDITEELSQ<br>HGQIAEQNVYNWFQNRRARSKRKQHGGGSSGNNN[illegible]SEVETEVEALNEKRVVRPESLLGLPDGNSNN<br>NGLGTTTATTTAPRPEDLCFQSPEISSDLHLLDVLSN[1]**P**RDEHLVGKMGLAESYNLYDHVEDYGMSG
X	MVKKKKEKEKSKEIEEMDREIQNGAYSGRVMTEEQMEILRKQIAVYAVICDQLVLLHNSLSSYHPLSS[1]**G**<br>VRPMVGGYFDPMGASSSSHRISTRHRWTPTSTQLQILESIYDEGSGTPNRRRIREIATELSEHGQITETNV<br>YNWFQNRRARSKRKQPQTTTANGQADDVAVTTEERRSCGDSGGLESYEHILFPSPD[1]**L**GIEHLLSIGKFM<br>ET
XI	MASSNRHWPSMFKSKPHPHQWQHDINSPLLPSASHRSSPFSS[1]**G**CEVERSPEPKPRWNPKPEQIRILEAIF<br>NSGMVNPPREEIRRIRAQLQEYGQVGDANVFYWFQNRKSRSKHKLRLLHNHSKHSLPQTQPQPQPQPS<br>ASSSSSSSSSSSKSTKPRKSKNKNNTNLSLGGSQMMGMFPPEPAFLFPVSTVGGFEGITVSSQLGFLSGD<br>MIEQQKPAPTCTGLLLSEIMNGSVSYGTHHQQHLSEKEVEEMRMKMLQQPQTQICYATTNHQIASYNN<br>NNNNNNIMLHIPPTTSTATTITTSHSLATVPSTSDQLQV[1]**Q**ADARIRVFINEMELEVSSGPFNVRDAFGEEV<br>VLINSAGQPIVTDEYGVALHPLQHGASYYLI
XII	MDQEQTPHSPTRHSRSPPSSASGSTSAEPVRSRWSPKPEQILILESIFHSGMVNPPKEETVRIRKMLEKFG<br>AVGDANVFYWFQNRRSRSRRRQRQLQAAAAAADATTNTCDQTMMVSNSLPHHSGSDLGFGGCSTSS<br>NYLFGGSSQVPSFFLGLSSSPSSCSSSSSTSSSASSSSSYGGGCDNQSNSGMENLLTMSGQMSYHEATHH<br>HYQNHSSNVTSILCPSDQNSNFQYQ[1]**Q**GAITVFINGVPTEVTRGGIDMKATFGEDLVLVHSSGVPLPTDEF<br>GFLMHSLQHGEAYF[0]**L**VPRQT

Note: I-VIII belong to the Modern clade; IX and X belong to the Intermediate clade; XI and XII belong to the Ancient clade. The residue in red indicates the intron insertion site, and the number besides it means the phase (0, 1, and 2) of the corresponding intron.
