# Supplementary material for: Global Analysis of WOX Transcription Factor Gene Family in Brassica napus Reveals Their Stress- and Hormone-Responsive Patterns
Source: Int J Mol Sci. 2018 Nov 5;19(11):3470. doi: 10.3390/ijms19113470 (PMC6274733; doi:10.3390/ijms19113470)
Supplement: Supplementary file 1 [file ijms-19-03470-s001.zip › ijms-372054-SI/Table S4.pdf]

Table S4. List of the 11 putative motifs of BnWOX proteins by MEME

Motif	E-value	Length	Sequence	Clade or subclade
Motif 1	9.60E-107	33	HYNHHRPYDHMSFACCSQPSPICLSHQAIGVEA	WUS, WOX1, WOX2 and WOX3 subclades
Motif 2	1.40E-124	50	LVSNHGFDKKDPPGYKVEQTKNWICSVGCDTQPZKPHEHHHQEEPVSIAL	WOX1 subclade
Motif 3	5.20E-166	21	QPJPTDEFGFLMHSLQHGASY	Intermediate clade
Motif 4	1.50E-106	29	GADSFNGGRKLRPLIPRLSSCPSSPANTN	WOX4 and WOX1subclades
Motif 5/acidic region	--	9	EEEECGGDA	WUS subclade
Motif 6	5.3E-392	29	VVMTGEQMEVLRKQIAMYATICEDLVLLH	Ancient clade, WOX11/12 subclade
Motif 7	7.90E-97	21	NNGMENLFTMYGHESDHEHLHHHYSN	WOX11/12 subclade
Motif 8/WUS-box	2.00E-185	15	IVIKTLELFPLHPED	WUS clade
Motif 9	3.10E-100	33	ASSNGYMSSHLYGSMEQDCSMSYNNVGGGWTNMDH	WUS subclade
Motif 10/HB	2.8E-2073	50	RTPSADZIQRITAELSKYGKIEGKNVFYWFQNHKARERQKQRQLQAASAA	Modern, Ancient and Intermediate clades
Motif11/EAR-like	--	5	LXLXL	WOX5 and WUS subclades

Note: --indicates the motif was identified by multiple alignment analysis using MAFFT.
